# A Diamine‐Oriented Biorefinery Concept Using Ammonia and Raney Ni as a Multifaceted Catalyst

**DOI:** 10.1002/cite.202200091

**Published:** 2022-09-19

**Authors:** Xianyuan Wu, Mario De bruyn, Katalin Barta

**Affiliations:** ^1^ University of Groningen Stratingh Institute for Chemistry Groningen The Netherlands; ^2^ University of Graz Department of Chemistry, Organic and Bioorganic Chemistry Heinrichstraße 28/II 8010 Graz Austria

**Keywords:** Amine, Catalysis, Cellulose, Lignin

## Abstract

Diamines are important industrial chemicals. In this paper we outline the feasibility of lignocellulose as a source of diol‐containing molecules. We also illustrate the possibility of turning these diols into their diamines in good to excellent yields. Central to these transformations is the use of commercially available Raney Ni. For diol formation, the Raney Ni engages in hydrogenation and often also demethoxylation, that way funneling multiple components to one single molecule. For diamine formation, Raney Ni catalyzes hydrogen‐borrowing mediated diamination in the presence of NH_3_.

## Graphical abstract

This work describes a novel sustainable biorefinery strategy, through which a range of biomass‐derived diols and high value diamines can be produced in excellent yields. Central to this is the use of Raney Ni, which engages in demethoxylation, hydrogenation, and amination, the latter following a hydrogen borrowing strategy.

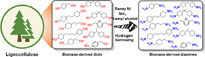

## 1 Introduction

Primary diamines hold vast industrial importance as prime building blocks for the polymer and pharmaceutical industry 1, 2, 3, 4. More specifically, they are widely used as monomers for the synthesis of amides (e.g., Nylon) 5, 6, polyurethanes 7, 8, 9, polyimides 10, 11, or as crosslinkers, curing agents, and hardeners in the synthesis of epoxy resins 12, 13, and polybenzoxazines 14, 15. Following suitable reductive alkylation, diamines can also serve as valuable intermediates for the pharmaceutical industry 16, switchable solvents 17, and as precursors to (hetero)gemini surfactants 18.

**Figure 1:**
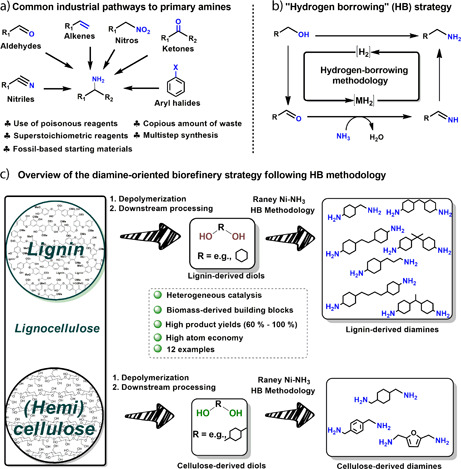
Overview of the “diamine‐oriented biorefinery” concept. A) Common industrial pathways to primary amines; B) general reaction mechanism of the HB strategy for the conversion of diols to diamines; C) general overview of a comprehensive downstream catalytic processing methodology converting lignocellulose‐derived diols to diamines.

Various methods have been described to the formation of primary amines, many of which are in principle also applicable to the synthesis of diamines. Commonly industrial processes to the manufacturing of primary amines follow a) N‐introduction via ‐CN or ‐NO_2_ groups, followed by hydrogenation 19, 20, b) reductive amination starting from ketones/aldehydes 21, c) alkene hydroformylation followed by amination and hydrogenation 22, d) direct alkene amination 21, e) Buchwald‐Hartwig amination with aryl halides (Fig. 1A). With most synthetic amination methodologies involving petroleum‐derived substrates, and featuring multistep synthetic routes, a high demand exists for the development of efficient catalytic methodologies that use renewable resources 23, 24, 25, 26, 27. Due to the abundant presence of hydroxy groups in biomass‐derived compounds, catalytic amination by the hydrogen‐borrowing (HB) methodology offers a great way to the introduction of nitrogen. More specifically, the HB amination methodology comprises the dehydrogenation of an alcohol functionality, followed by intermediate imine formation, and final imine hydrogenation towards the target amines 28, 29, 30. Notably, primary amines are obtained when ammonia is used as a coupling partner to the alcohols, this transformation being particularly challenging as outlined below 31, 32, 33. Key benefits to the HB amination methodology are the non‐requirement of pressurized H_2_ gas and the fact that water is the only by‐product (Fig. 1B). Over recent years the HB amination field has seen significant progression with the development of a range of homogeneous 28 and heterogeneous 29 catalytic systems. Notable examples to the heterogeneous catalytic amination of alcohols to primary amines via HB are: Ni/*θ*‐Al_2_O_3_ (or Ni/γ‐Al_2_O_3_) 34, Ni/Al_2_O_3_‐SiO_2_ 35, Ni/CaSiO_3_ 36, Raney Ni 37, NiAl/HT 38, Pd/CeO_2_ 39, and PtCo/CeO_2_ 40.

A major challenge to HB‐mediated amination with ammonia is the undesired formation of secondary and tertiary amines, suppression of which typically requires higher ammonia excesses 37. In the gas phase this equals to the use of very high ammonia pressures (>300 bar), therewith explaining its current industrial redundancy 41. Catalyst deactivation has also been observed and this especially with non‐noble metal catalysts 42. For example, it was found that NH_3_ preferably reacts with Ni to form catalytically inactive Ni_3_N for the reductive amination of phenol to cyclohexylamine 42. It is equally noteworthy that HB amination of (primary) diols tends to lead to incomplete amination (i.e., a mixture of amino alcohols) thus posing severe separation challenges [43,44].

Irrespective the exact amination mechanism, a limited number of heterogeneous catalytic procedures for diol‐diamination have been reported. Moreover, these concern quasi always single substrates and almost all developed methodologies employ a positive H_2_ pressure as to suppress undesired dehydrogenation. Exemplary heterogeneous catalytic systems to the diol‐to‐diamine transformation are:

1. The Ru/MgO‐TiO_2_ catalyzed amination of 2,5‐bis(hydroxymethyl)furan into 2,5‐bis(aminomethyl)‐furan in 86 % yield through an HB strategy 45.
2. The conversion of isomannide to a mixture of mono‐ and diamines using Ru/C in respectively 40 and 10 % yield using more than 10 bar of H_2_ 41. Through this methodology biogenic diols (e.g., isosorbide, isomannide) were generally observed to form predominately amino alcohols over diamines, which was attributed to the preferential adsorption of formed diamines on the active sites, therewith blocking these 41.
3. The catalytic amination of 1,3‐propanediol to 1,3‐propanediamine in supercritical NH_3_ using a CoFe alloy in 32.2 % yield, in the presence of a small amount of H_2_ 46.
4. The direct amination of 1,4‐butanediol over a Cu, Ni, Ti, Zr, Sn, Co, Mn/zeolite catalyst to afford 1,4‐butanediamine in 70 % yield using an NH_3_/H_2_ mixture with a respective 3:1 ratio 47, 48.
5. The Raney Ni catalyzed diamination of benzenedimethanols to xylylenediamines (para yield: 63 %; meta yield: 50 %), as reported by our group 37. This methodology was recently extended to the quantitative conversion of 4,4‐methylenebiscyclohexanol into its corresponding diamine 44.

In this paper we present a range of strategies for the development of a potential diamine‐oriented biorefinery, thereby considering various cutting‐edge lignin depolymerization strategies as well as the valorization of the cellulose platform. Naturally, the proposed strategies could in the future be combined with other established biorefinery schemes that may already focus on different product classes such as biofuels or materials. In our current discussion, neither routes to fuels nor the manufacturing of complex specialty chemicals were considered, but rather, full focus was devoted to the synthesis of diamines. To this purpose this paper first highlights potential routes towards suitable diols from key lignin and cellulose‐derived platform chemicals focusing on the use of Raney Ni as an industrially relevant catalyst, and this in line with two previous topical papers by our group 37, 44. Interestingly, especially for the lignin platform, Raney Ni is responsible for key hydrogenation and demethoxylation steps to afford a single diol, even from more complex bio‐oil mixtures. This builds further on a topical diamination paper recently published by our group 37. The current paper exemplifies the broad applicability of this methodology, and the high achievable isolated diamine yields over a wide range of compounds (Fig. 1C).

## 2 Results and Discussion

### 2.1 Catalytic Strategies to Access Well‐Defined Lignin‐Derived Diols

Due to the parent monolignols from which lignin biosynthesis occurs, guaiacyl (G) and syringyl (S) units are the dominant structural moieties in lignin and this consequently also in the platform chemicals obtained by various depolymerization methods. These structures feature neighboring aromatic alcohol and methoxy groups and offer vast potential for the transformation into (aliphatic) alcohols. This is not only achievable by the creation of 1,2‐diol motifs following demethylation/(hydrogenation) of guaiacyl units 49, 50 but also by consecutive demethoxylation and hydrogenation of G‐ and S‐containing aromatic moieties into their corresponding aliphatic alcohols 51. In line with previous investigations by our group, the use of such tandem defunctionalization/hydrogenation steps is one of the main foci of this paper.

**Figure 2:**
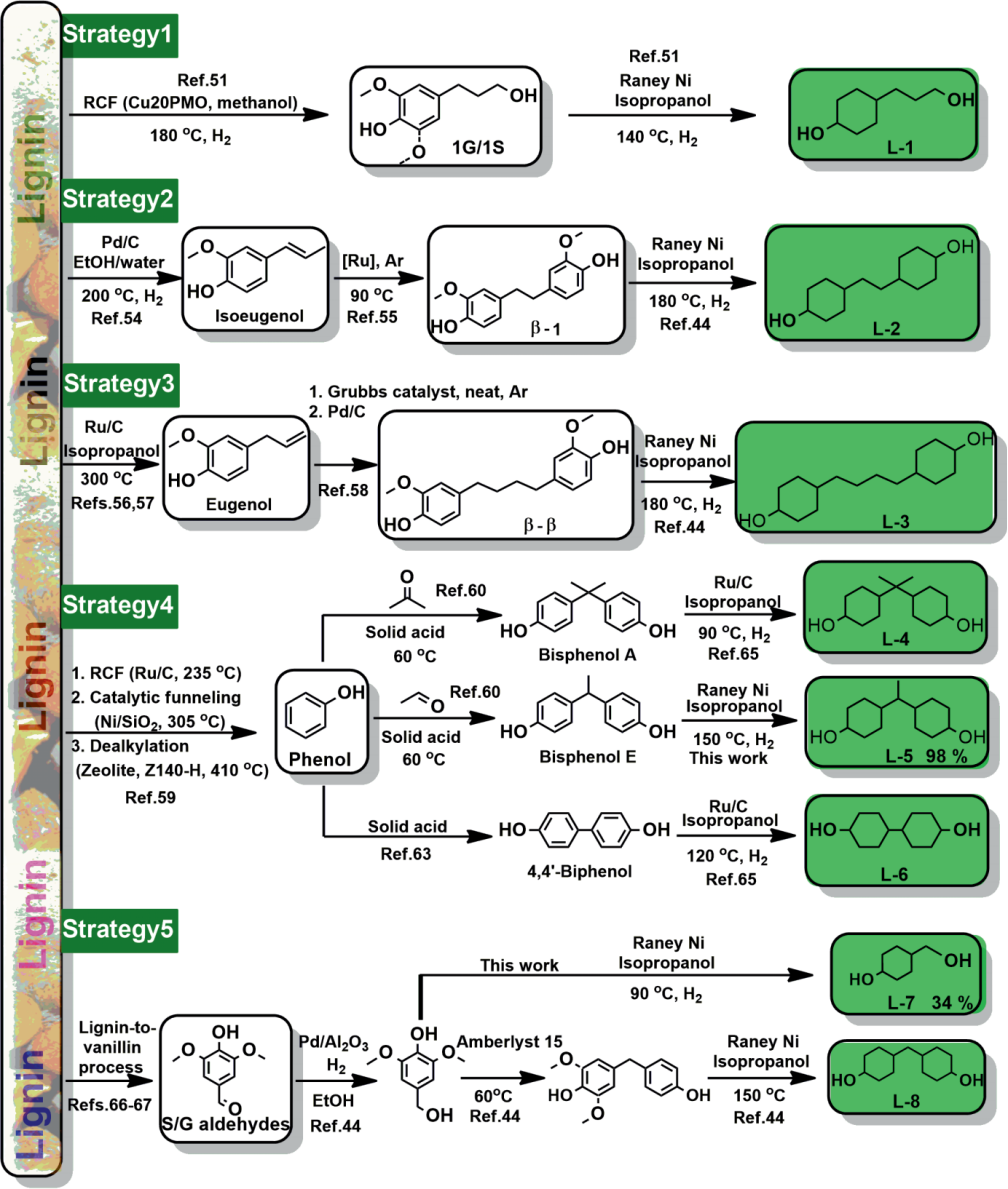
Overview of a range of catalytic strategies to turn lignin‐derived platform chemicals into cyclic aliphatic diols.

Key aspects to consider when aiming for efficient catalytic conversion strategies from lignin to diols are: 1) the lignin depolymerization method; 2) the structural nature and reactivity of the lignin‐derived platform chemicals and 3) suitable downstream processing strategies. Herein we detail a range of specific strategies (Strategy 1–5), taking our own findings, and the ones of other groups, as suitable starting points. Reductive catalytic fractionation (RCF) is a central method for the efficient conversion of native lignin into aromatic platform chemicals. RCF typically entails the use of H_2_ or an (in)organic H‐donor and a heterogeneous transition metal catalyst, which is introduced during the fractionation process. The process conditions, type of lignocellulose and type of catalyst have significant impact on the product outcome. For example, when Cu20PMO is used for RCF of lignocellulosic biomass, monomeric dihydroconyferyl alcohol (1G) and/or dihydrosynapyl alcohol (1S) are obtained as the main products 52. Conversely, the use of noble metals, and this typically at higher operational temperatures (≥ 250 °C), results in the formation of 4‐propyl guaiacol/syringol 53. Accordingly, several specific strategies can be developed for obtaining diols based on the RCF methodology (Strategy 1–4):

*Strategy 1*: Cu20PMO‐created 1G and 1S can be subjected to demethoxylation and hydrogenation to yield diol **L‐1** over Raney Ni catalyst at 150 °C (Fig. 2). This procedure is particularly interesting as it concerns funneling catalysis whereby both guaiacyl and syringyl units are converted into the **L‐1** compound 51.

*Strategy 2* concerns the valorization of isoeugenol, a compound which can be obtained by Pd‐catalyzed transfer hydrogenolysis of pine wood lignin using EtOH/water at 195 °C 54. Self‐metathesis of isoeugenol to *β*‐1 bisphenol 55, followed by demethoxylation and hydrogenation with Raney Ni at 180 °C, yields diol **L‐2** 44.

*Strategy 3* entails the catalytic transfer hydrogenolysis (Ru/C) of ionic liquid processed biorefinery lignin in the presence of isopropanol – an economically viable way to eugenol 56, 57. Solvent‐free self‐metathesis of eugenol (over a Grubbs catalyst) and hydrogenation (Pd/C) leads to the formation of *β–β* bisphenol 58, a compound which can be further demethoxylated and hydrogenated with Raney Ni at 180 °C to **L‐3** 44.

*Strategy 4* constitutes a three‐step catalytic methodology to lignin‐derived phenol in 20 wt % yield as developed by the Sels group: 1) Ru/C catalyzed reductive lignin depolymerization, 2) Ni/SiO_2_‐mediated selective removal of the methoxy groups therewith generating 4‐alkylphenols and 3) dealkylation by zeolite Z140‐H to phenol 59. Then, condensation of two molecules of phenol with respectively acetone and acetaldehyde, in the presence of a strong solid acidic catalyst 60 or simply HCl 61, gives bisphenol‐A and bisphenol‐E (Fig. 2). In spite of the latter being a homogeneous acid, it is noteworthy that it is inexpensive, widely used industrially, and recyclable 62. Next to bisphenols, phenol can also be dimerized into 4,4'‐biphenol over a solid acid catalyst (≥ 225 °C) 63. Alternatively, it was also recently shown that 1 wt % Au/TiO_2_ can catalyze the dimerization of phenol to biphenol at 150 °C in the presence of 12 bar O_2_, potentially opening the door to a fully sustainable process 64. The subsequent hydrogenation of the aromatic ring in bisphenols and biphenol can be achieved by our Raney Ni or Ru/C methodology at 90–150 °C 65, yielding diols **L‐4**, **L‐5** and **L‐6**.

In addition to diols obtained from the RCF pathway, the oxidative depolymerization of lignin, a methodology which tends to yield larger amounts of vanillin, can also be used to create diols. For example, *Strategy 5* describes the formation of diol **L‐7** (34 % yield) from vanillin over vanillyl alcohol over Raney Ni catalyst at 90 °C – the route also holding for the syringyl equivalents of the latter two compounds. Even though the direct chemical vanillin production from lignin has been in stark decline – the Borregaard company being the last industrial manufacturer following that route 66, 67 – it must be noted that the main worldly vanillin production method does still start from guaiacol, a compound relatively easily derivable from lignin 68, 69. Over the last two decades though, also a range of biotechnological routes to vanillin have become commercial realities, the starting materials being lignin, ferulic acid, curcumin (turmeric), and eugenol/isoeugenol 70. Of particular note is also the fact that the waste streams of the paper and pulp industry are excellent resources to the making of benzaldehydes, among which vanillin. Another possibility to derive diols from vanillin is by reducing vanillin (or its syringyl equivalent) to its corresponding benzylic alcohol, which upon dehydration react readily with phenol following an electrophilic aromatic substitution 44. The so resulting diaromatic products can be demethoxylated/hydrogenated by Raney Ni to **L‐8** 44 (Fig. 2).

### 2.2 Catalytic Strategies to Well‐Defined Cellulose‐Derived Diols

A long‐time central platform molecule from cellulose is 5‐hydroxymethylfurfural (HMF) – many cellulose‐based methods to which have been developed 71, 72, 73. Three main strategies exist to convert HMF to diols:

*Strategy A* consists of the controlled reduction of HMF to **C1** 74, 75, 76, 77, 78, 79, 80, 81, 82, a transformation to which many catalytic methods have been reported. We have previously reported on the use of a Cu‐Zn alloy nanopowder catalyst in ethanol at 120 °C and 70 bar H_2_ 81 and a Cu20PMO catalyst in ethanol at 120 °C and 50 bar H_2_ 82. Other notable examples include the use of Pt/MCM‐41 83, Pt/CeO_2_‐ZrO_2_ 84, PtSn/Al_2_O_3_ 85 and Ir‐ReOx/SiO_2_ 86 at mild reaction conditions 87.

*Strategy B* involves the transformation of HMF to dimethylfuran (DMF) and further on, via Diels‐Alder reaction with ethylene and subsequent acid‐catalyzed ring opening/dehydration, to *p*‐xylene 88, 89, 90, 91. To this end Tao et al. proposed a most interesting one‐pot procedure to produce DMF from HMF, based on a Pd‐Au/ZrO_2_ catalyst and the use of formic acid as the H‐donor 91. The conversion of *p*‐xylene to **C2** can be achieved at very mild temperature (10 °C) using photo‐bromination to 1,4‐bis(bromomethyl)benzene (BBMB), followed by a substitution reaction with an alkali base/carboxylate compound 92. Alternatively, *p*‐xylene can undergo direct oxidation into *p*‐terepththalic acid (TPA) via the Amoco process, the currently industrially practiced way. More specifically, the Amoco process concerns the oxidation of 25 % *p*‐xylene in glacial acid at 175–225 °C and 15–30 bar air in the presence of manganese and cobalt salts as catalysts, and a bromide salt as a promotor, and this over a total reaction time of 1 h 93. In turn TPA can be hydrogenated to compound **C2** using Ru/Sn/B supported on Al_2_O_3_ (230 °C, 100 bar H_2_) 94. The subsequent hydrogenation of **C2** into **C3** can be achieved in 91.2 % yield using a Ru/Al_2_O_3_ catalyst in water (100 °C, 80 bar H_2_) 95. A 2014 patent reports the (rare) direct hydrogenation of TPA to **C‐3** in 42 % yield using a Ru/Sn‐Re catalyst at 250 °C and 150 bar H_2_ 94.

*Strategy C:* Several pathways were also reviewed to produce TPA from cellulose‐derived platform compounds 96. One important way is the transformation of HMF into furan‐2,5‐dicarboxylic acid (FDCA), a process which is most commonly performed using heterogeneous catalysts in the presence of an excess of O_2_ or air 97, 98. Very recently though it was shown that a homogeneous ruthenium pincer catalyst could perform this oxidation using alkaline water as the formal oxidant and producing H_2_ in the process 99. With though two electron‐withdrawing substituents on the furan, FDCA is poorly reactive in the Diels‐Alder reaction with ethylene to TPA 89. The currently most performing catalytic methods to this transformation are: 1) the use of an aluminum‐pillared montmorillonite at 250 °C and 60 bar ethylene (50 % TPA yield) and 2) silica‐supported heteropolyacid at 225 °C and 30 bar ethylene (60 % TPA yield) 89. The obtained TPA can be further converted to diols **C2** and **C3** as described above.

**Figure 3:**
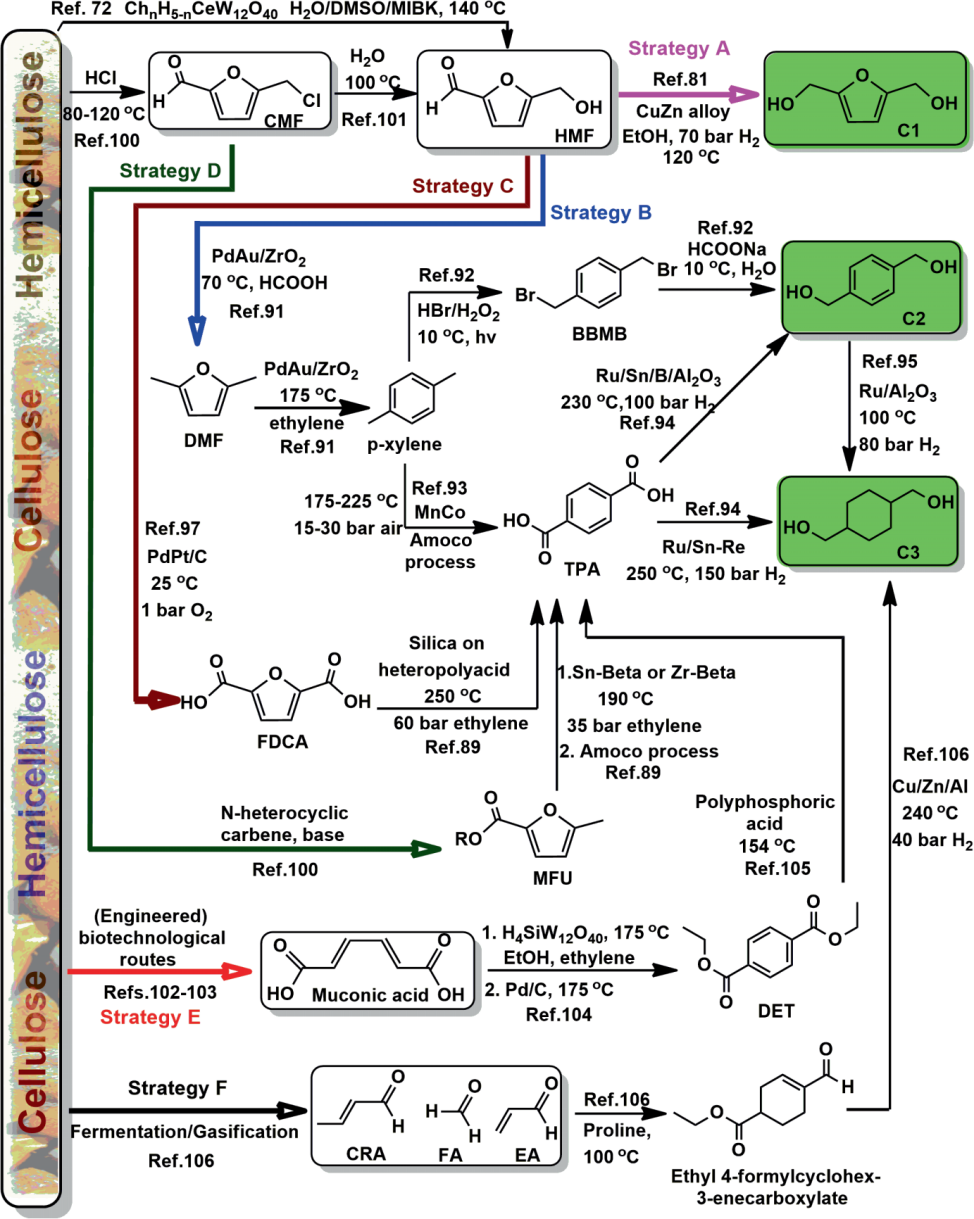
Overview of a range of catalytic strategies to turn (hemi)cellulose‐derived platform chemicals into cyclic aliphatic/aromatic diols.

In addition to diols obtained from HMF, other cellulose‐derived platform chemicals can also serve as suitable starting materials for diols (Fig. 3), exemplary being:

*Strategy D* proposes the production of **C3** starting from CMF, a more stable analogue/alternative of HMF, which can be synthesized from cellulose using aqueous HCl in a biphasic reaction in between 80–120 °C 100. The direct synthesis of 5‐methyl‐2‐furanoate (MFU) from CMF can be achieved via a redox‐neutral N‐heterocylic carbene catalyst in the presence of base 100. Then TPA can be obtained through a Diels‐Alder reaction with ethylene (35 bar) in the presence of Lewis acidic zeolites (e.g., Sn‐Beta, Zr‐beta) as the catalyst at 190 °C 89, followed by the Amoco process (see before). Interestingly, CMF can also be directly converted to 5‐HMF in water at 100 °C 101.

*Strategy E* details the conversion of muconic acid to TPA. Muconic acid can be obtained from saccharides through mainly (engineered) biotechnological routes 102, 103. In contrast to FDCA, the absence of aromaticity in muconic acid makes that it engages easier in Diels‐Alder reactions. Alike to the FDCA case, the muconic acid Diels‐Alder reaction is preferentially run from the muconic acid diester. Diels‐Alder reaction between (esterified) muconic acid and ethylene (high pressure) leads to the formation of the diesters of cyclohex‐2‐ene‐1,4‐dicarboxylic acid and cyclohex‐1‐ene‐1,4‐dicarboxylic acid, both of which can be converted to diethyl terephthalate (DET) in 80.6 % yield when using silicotungstic acid for the Diels‐Alder step (99 % yield) and Pd for the dehydrogenation reaction 104. TPA can be obtained by hydrogenolysis of DET in a yield of 96 % using polyphosphoric acid 105. It is noteworthy that muconic acid can also be obtained from lignin‐derived catechol (and by extension guaiacol and phenol) through chemical and biotechnological routes 103.

*Strategy F* yields **C3** via an unusual, yet highly interesting, pathway as published by the groups of Wang and Li 106. It concerns the formal [3+1+2] proline‐catalyzed condensation of crotonaldehyde (CRA), formaldehyde (FA) and ethyl acrylate (EA) to ethyl 4‐formylcyclohex‐3‐enecarboxylate, which is then converted to **C3** in 83 % yield (240 °C, 40 bar H_2_) using a commercial Cu/Zn/Al catalyst 106.

### 2.3 Catalytic Strategies to Well‐Defined Lignocellulose‐Derived Diamines

**Figure 4:**
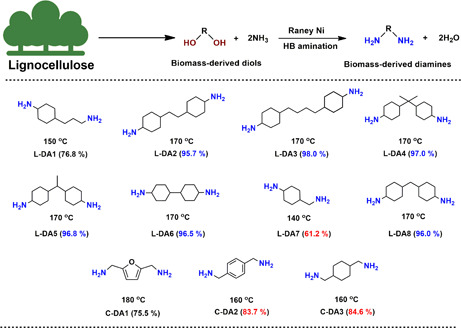
Diamination scope of lignocellulose‐derived diols. Reaction conditions: 0.5 mmol diols, 50 mg catalysts, 2.5 mL *t*‐amyl alcohol, 140–180 °C, 18 h, 10 mg dodecane as the internal standard; GC‐FID determined yields by means of an internal standard are shown in black; yields determined by means of a calibration curve are depicted red; Isolated yields are shown in blue.

Within this work we set out for the identification of a suitable catalytic amination strategy that can convert a wide variety of lignin‐ and cellulose‐derivable diols (Figs. 2 and 3) directly into diamines, and this preferentially using ammonia as the N‐donor and following the HB methodology. However, such a direct catalytic transformation of diols to diamines is generally significantly challenging with the achievable catalytic efficiency being low and the final purification protocol being tedious. To achieve this goal, we evaluated the use of Raney Nickel, a catalyst which our group has previously shown to be very efficient to the transformation of the industrially relevant diol **L‐8** into **L‐DA8** (96 % yield) and this using ammonia and an HB methodology. Using optimized reaction conditions 44, this technology proved to be applicable to all the above‐mentioned diol‐containing substrates therewith underscoring the power of this method for the development of diamine oriented biorefinery concepts (Fig. 4).

With **L‐1** and **L‐7** featuring both an aliphatic primary alcohol and a secondary alcohol, the possibility existed that these non‐equal alcohol functions would show a different catalytic coupling reactivity with ammonia. In this area, pioneering work by Shimizu and co‐workers has shown that cyclic aliphatic secondary alcohols are more prone to amination than are aromatic/aliphatic primary alcohols 34. When subjecting diol **L‐1** and **L‐7** to the optimized Raney Ni catalyzed diamination methodology, it is observed that all cyclic secondary alcohol moieties undergo fast dehydrogenation/amination/hydrogenation to primary amine while the primary alcohol groups convert much slower. It is exactly the latter observation that lays at the basis of the lower yield of **LDA‐1** and **LDA‐7**, 76.8 % and 61.2 %, respectively. In line with this observation diols **L‐2**, **L‐3**, **L‐4**, **L‐5**, **L‐6** and **L‐8**, which each bear two cyclic secondary alcohol groups, displayed a very high diamination reactivity, delivering the corresponding diamines (**LDA‐2**, **LDA‐3**, **LDA‐4**, **LDA‐5**, **LDA‐6** and **LDA‐8**) in 95.7–98 % isolated yield. Importantly, the latter diamines are all highly important monomers to the production of high‐performing polymers such as polybenzoxazine 44 and epoxy vitrimer 107.

As to the cellulose‐derivable diamines it is noteworthy that **CDA‐1** holds great potential to the construction of novel biopolymers. Recent reports on its synthesis have focused predominately on the heterogeneous/homogeneous catalytic reductive amination of 5‐HMF 108, 109, 2,5‐diformylfuran (DFF) 110, 5‐(chloromethyl)furfural (CMF) 111, 5‐(hydroxymethyl)furfurylamine 112, 113, 2,5‐dihydroxymethylfuran (**C1**) 45 or 2,5‐diformylfuran dioxime 114. For each of these approaches ammonia was used as the N‐donor and **CDA‐1** diamine was obtained in good to high yield. Applying our optimized Raney Ni/NH_3_ catalyzed diol amination method, **CDA‐1** could be obtained in 75.5 % overall yield, a most encouraging result.

**CDA‐2** is an industrially important diamine, widely used in the making of thermally stable polyamide fibers (e.g., Kevlar®) 37. In a previous study it was found possible to convert **C2** into **CDA‐2** at 180 °C using Raney Ni and aqueous NH_3_ in 61 % overall yield 34. Gratifyingly, the here employed Raney Ni methodology, which uses *t*‐amyl alcohol as the solvent and NH_3_ gas, and operates at a lower temperature 160 °C, increased the attainable yield to 83.7 %. Lastly, the deamination of diol **C3** was found to run smoothly, yielding **CDA‐3** in 84.6 % yield.

## 3 Conclusion

In this paper we have described in detail a potential diamine oriented biorefinery concept. Firstly, we considered high‐yielding pathways toward platform chemicals from lignin as well as cellulose that are suitable and ideally set up for further downstream processing to diols. Next, a straightforward atom‐economical methodology was described to convert these diols into their diamine equivalents. Central to these transformations stands the capability of Raney Ni to act as a hydrogenation, demethoxylation and hydrogen‐borrowing amination catalyst. The wide commercial availability of Raney Ni, as well as its vast industrial use, adds strongly to the potential viability of such a diamine‐oriented biorefinery concept. This is further underscored by the excellent diamine (isolated) yields, often exceeding 95 %.

## 4 Experimental

### 4.1 Materials and reagents

Chemicals were used as received, unless otherwise specified. Raney Ni was purchased from Sigma Aldrich.

The catalytic direct amination of diols into diamines was performed in a 10‐mL high pressure autoclave equipped with a magnetic stirring bar. Typically, a 4‐mL vial was charged with 50 mg Raney Ni catalyst, 0.5 mmol diol, 2.5 mL *t*‐amyl alcohol, 10 mg dodecane as an internal standard. Then the vial was put inside an autoclave and pressurized with 7 bar NH_3_. The reactor was heated and stirred at 400 rpm for 18 h. After completion of the reaction, the reactor was cooled down to room temperature. Then, 0.1 mL solution was collected through a syringe and injected in a GC‐MS or GC‐FID after filtration through a PTFE filter (0.45 µm).

### 4.2 Characterizations

NMR spectroscopy: At the University of Groningen, ^1^H, ^13^C NMR and 2D NMR spectra were recorded on a Varian Mercury Plus 400, Agilent MR 400 and a Bruker Avance NEO 600 (600 and 151 MHz, respectively); At the University of Graz: ^1^H, and ^13^C NMR spectra were recorded on a Bruker Avance III 300MHz and 2D NMR spectra were recorded on a Bruker Avance III 700MHz equipped with a 5 mm Triple‐Resonance cryoprobe.

## Supporting Information

Supporting Information for this article can be found under DOI: https://doi.org/10.1002/cite.202200091.

## Abbreviations

L‐1: 4‐(3‐hydroxypropyl)cyclohexanol
L‐2: 4,4′‐(ethane‐1,2‐diyl)dicyclohexanol
L‐3: 4,4′‐(butane‐1,4‐diyl)dicyclohexanol
L‐4: 4,4′‐(propane‐2,2‐diyl)dicyclohexanol
L‐5: 4,4′‐(ethane‐1,1‐diyl)dicyclohexanol
L‐6: [1,1′‐bi(cyclohexane)]‐4,4′‐diol
L‐7: 4‐(hydroxymethyl)cyclohexanol
L‐8: 4,4′‐methylenedicyclohexanol
C1: 2,5‐bis(hydroxymethyl)furan
C2: 1,4‐benzenedimethanol
C3: 1,4‐cyclohexanedimethanol
LDA‐1: 4‐(3‐aminopropyl)cyclohexanamine
LDA‐2: 4,4′‐(ethane‐1,2‐diyl)dicyclohexanamine
LDA‐3: 4,4′‐(butane‐1,4‐diyl)dicyclohexanamine
LDA‐4: 4,4′‐(propane‐2,2‐diyl)dicyclohexanamine
LDA‐5: 4,4′‐(ethane‐1,1‐diyl)dicyclohexanamine
LDA‐6: [1,1′‐bi(cyclohexane)]‐4,4′‐diamine
LDA‐7: 4‐(aminomethyl)cyclohexanamine
LDA‐8: 4,4′‐methylenedicyclohexanamine
CDA‐1: 2,5‐bis(aminomethyl)furan
CDA‐2: 1,4‐benzenedimethanamine
CDA‐3: 1,4‐bis(aminomethyl)cyclohexane
β‐1: 4,4′‐(ethane‐1,2‐diyl) bis(2‐methoxyphenol)
β–β: 4,4′‐(butane‐1,4‐diyl) bis(2‐methoxyphenol)

## Supporting information

Supplementary Information

## References

[1] V. Froidevaux , C. Negrell , S. Caillol , J. P. Pascault , B. Boutevin , Chem. Rev. 2016, 116 (22), 14181–14224. DOI: 10.1021/acs.chemrev.6b00486 27809503

[2] Z. H. Sun , B. Fridrich , A. de Santi , S. Elangovan , K. Barta , Chem. Rev. 2018, 118 (2), 614–678. DOI: 10.1021/acs.chemrev.7b00588 29337543 PMC5785760

[3] M. Pelckmans , T. Renders , S. Van de Vyver , B. F. Sels , Green Chem. 2017, 19 (22), 5303–5331. DOI: 10.1039/C7GC02299A

[4] J. He , L. L. Chen , S. M. Liu , K. Song , S. Yang , A. Riisager , Green Chem. 2020, 22 (20), 6714–6747. DOI: 10.1039/D0GC01869D

[5] M. Winnacker , B. Rieger , Macromol. Rapid. Commun. 2016, 37 (17), 1391–1413. DOI: 10.1002/marc.201600181 27457825

[6] Y. Jiang , D. Maniar , A. J. J. Woortman , G. O. R. A. van Ekenstein , K. Loos , Biomacromolecules 2015, 16 (11), 3674–3685. DOI: 10.1021/acs.biomac.5b01172 26418272

[7] G. Rokicki , P. G. Parzuchowski , M. Mazurek , Polym. Advan. Technol. 2015, 26 (7), 707–761. DOI: 10.1002/pat.3522

[8] O. Figovsky , L. Shapovalov , A. Leykin , O. Birukova , R. Potashnikova , Chem. Chem. Techol. 2013, 7 (1), 79–87. DOI: 10.23939/chcht07.01.079

[9] K. Blażek , J. Datta , Crit. Rev. Environ. Sci. Technol. 2019, 49 (3), 173–211. DOI: 10.1080/10643389.2018.1537741

[10] A. S. Hicyilmaz , A. C. Bedeloglu , SN Appl. Sci. 2021, 3 (3), 363. DOI: 10.1007/s42452-021-04362-5

[11] P. C. Ma , C. T. Dai , H. Z. Wang , Z. K. Li , H. B. Liu , W. M. Li , C. L. Yang , Compos. Commun. 2019, 16, 84–93. DOI: 10.1016/j.coco.2019.08.011

[12] E. A. Baroncini , S. Kumar Yadav , G. R. Palmese , J. F. Stanzione III , J. Appl. Polym. Sci. 2016, 133 (45), 44103. DOI: 10.1002/app.44103

[13] F. S. Hu , S. K. Yadav , J. J. La Scala , J. M. Sadler , G. R. Palmese , Macromol. Chem. Phys. 2015, 216 (13), 1441–1446. DOI: 10.1002/macp.201500142

[14] G. Lligadas , A. Tuzun , J. C. Ronda , M. Galia , V. Cadiz , Polym. Chem. 2014, 5 (23), 6636–6644. DOI: 10.1039/c4py00914b

[15] J. K. Liu , L. Y. Zhang , W. L. Y. Shun , J. Y. Dai , Y. Y. Peng , X. Q. Liu , J. Polym. Sci. 2021, 59 (14), 1474–1490. DOI: 10.1002/pol.20210328

[16] E. Bogatcheva , C. Hanrahan , B. Nikonenko , R. Samala , P. Chen , J. Gearhart , F. Barbosa , L. Einck , C. A. Nacy , M. Protopopova , J. Med. Chem. 2006, 49 (11), 3045–3048. DOI: 10.1021/jm050948+ 16722620 PMC4869334

[17] J. R. Vanderveen , J. L. Geng , S. Zhang , P. G. Jessop , RSC Adv. 2018, 8 (48), 27318–27325. DOI: 10.1039/c8ra05751f 35540014 PMC9083370

[18] B. E. Brycki , I. H. Kowalczyk , A. Szulc , O. Kaczerewska , M. Pakiet , Application and Characterization of Surfactants 2017, 97–155. 10.5772/intechopen.68755

[19] P. Roose , K. Eller , E. Henkes , R. Rossbacher , H. Höke , in Ullmann's Encyclopedia of Industrial Chemistry, Wiley‐VCH, Weinheim 2015, 1–50.

[20] S. A. Lawrence , Amines: Synthesis, Properties and Applications, Cambridge University Press, Cambridge 2005.

[21] K. S. Hayes , Appl. Catal., A 2001, 221 (1–2), 187–195. DOI: 10.1016/S0926-860X(01)00813-4

[22] S. Fuchs , D. Lichte , T. Jolmes , T. Rosler , G. Meier , H. Strutz , A. Behr , A. J. Vorholt , ChemCatChem 2018, 10 (18), 4126–4133. DOI: 10.1002/cctc.201800950

[23] B. M. Stadler , C. Wulf , T. Werner , S. Tin , J. G. de Vries , ACS Catal. 2019, 9 (9), 8012–8067. DOI: 10.1021/acscatal.9b01665

[24] X. Wang , S. Y. Gao , J. Wang , S. Xu , H. Li , K. Q. Chen , P. K. Ouyang , Chin. J. Chem. Eng. 2021, 30, 4–13. DOI: 10.1016/j.cjche.2020.12.009

[25] X. Chen , S. Song , H. Y. Li , G. Gozaydin , N. Yan , Acc. Chem. Res. 2021, 54 (7), 1711–1722. DOI: 10.1021/acs.accounts.0c00842 33576600

[26] S. S. Wong , R. Y. Shu , J. G. Zhang , H. C. Liu , N. Yan , Chem. Soc. Rev. 2020, 49 (15), 5510–5560. DOI: 10.1039/D0CS00134A 32639496

[27] K. Murugesan , T. Senthamarai , V. G. Chandrashekhar , K. Natte , P. C. J. Kamer , M. Mi , R. V. Jagadeesh , Chem. Soc. Rev. 2020, 49 (17), 6273–6328. DOI: 10.1039/C9CS00286C 32729851

[28] A. Corma , J. Navas , M. J. Sabater , Chem. Rev. 2018, 118 (4), 1410–1459. DOI: 10.1021/acs.chemrev.7b00340 29319294

[29] K.‐i. Shimizu , Catal. Sci. Technol. 2015, 5 (3), 1412–1427. DOI: 10.1039/c4cy01170h

[30] B. G. Reed‐Berendt , D. E. Latham , M. B. Dambatta , L. C. Morrill , ACS Cent. Sci. 2021, 7 (4), 570–585. DOI: 10.1021/acscentsci.1c00125 34056087 PMC8155478

[31] D. Pingen , C. Muller , D. Vogt , Angew. Chem., Int. Ed. 2010, 49 (44), 8130–8133. DOI: 10.1002/anie.201002583 20672264

[32] S. Imm , S. Bahn , L. Neubert , H. Neumann , M. Beller , Angew. Chem., Int. Ed. 2010, 49 (44), 8126–8129. DOI: 10.1002/anie.201002576 20677295

[33] C. Gunanathan , D. Milstein , Angew. Chem., Int. Ed. 2008, 47 (45), 8661–8664. DOI: 10.1002/anie.200803229 18846519

[34] K.‐i. Shimizu , K. Kon , W. Onodera , H. Yamazaki , J. N. Kondo , ACS Catal. 2013, 3 (1), 112–117. DOI: 10.1021/cs3007473

[35] A. Y. K. Leung , K. Hellgardt , K. K. Hii , ACS Sustainable Chem. Eng. 2018, 6 (4), 5479–5484. DOI: 10.1021/acssuschemeng.8b00338

[36] K.‐i. Shimizu , S. Kanno , K. Kon , S. M. A. H. Siddiki , H. Tanaka , Y. Sakata , Catal. Today 2014, 232, 134–138. DOI: 10.1016/j.cattod.2013.09.002

[37] Y. Z. Liu , A. Afanasenko , S. Elangovan , Z. H. Sun , K. Barta , ACS Sustainable Chem. Eng. 2019, 7 (13), 11267–11274. DOI: 10.1021/acssuschemeng.9b00619 31304071 PMC6614922

[38] K. Zhou , R. H. Xie , M. T. Xiao , D. R. Guo , Z. D. Cai , S. M. Kang , Y. J. Xu , J. J. Wei , ChemCatChem 2021, 13 (8), 2074–2085. DOI: 10.1002/cctc.202001922

[39] Z. Yan , A. Tomer , G. Perrussel , M. Ousmane , B. Katryniok , F. Dumeignil , A. Ponchel , A. Liebens , M. Pera‐Titus , ChemCatChem 2016, 8 (21), 3347–3352. DOI: 10.1002/cctc.201600855

[40] T. Tong , W. J. Guo , X. H. Liu , Y. Guo , C. W. Pao , J. L. Chen , Y. F. Hu , Y. Q. Wang , J. Catal. 2019, 378, 392–401. DOI: 10.1016/j.jcat.2019.08.024

[41] R. Pfutzenreuter , M. Rose , ChemCatChem 2016, 8 (1), 251–255. DOI: 10.1002/cctc.201501077

[42] T. Cuypers , T. Morias , S. Windels , C. Marquez , C. Van Goethem , I. Vankelecom , D. E. De Vos , Green Chem. 2020, 22, 1884–1893. DOI: 10.1039/C9GC02625H

[43] S. Imm , S. Bahn , M. Zhang , L. Neubert , H. Neumann , F. Klasovsky , J. Pfeffer , T. Haas , M. Beller , Angew. Chem., Int. Ed. 2011, 50 (33), 7599–7603. DOI: 10.1002/anie.201103199 21732514

[44] X. Y. Wu , M. V. Galkin , K. Barta , Chem Catal. 2021, 1 (7), 1466–1479. DOI: 10.1016/j.checat.2021.10.022

[45] Y. Kita , M. Kuwabara , S. Yamadera , K. Kamata , M. Hara , Chem. Sci. 2020, 11 (36), 9884–9890. DOI: 10.1039/d0sc03858j 34094248 PMC8162067

[46] A. Fischer , M. Maciejewski , T. Burgi , T. Mallat , A. Baiker , J. Catal. 1999, 183 (2), 373–383. DOI: 10.1006/jcat.1999.2408

[47] Y. Sun , J. Xu , J. Gao , X. Zheng , W. Du , S. Shi , *Patent* , 2016. CN105503613B

[48] B. M. Stadler , C. Wulf , T. Werner , S. Tin , J. G. de Vries , ACS Catal. 2019, 9 (9), 8012–8067. DOI: 10.1021/acscatal.9b01665

[49] J. Bomon , M. Bal , T. K. Achar , S. Sergeyev , X. Wu , B. Wambacq , F. Lemiere , B. F. Sels , B. U. W. Maes , Green Chem. 2021, 23 (5), 1995–2009. DOI: 10.1039/d0gc04268d

[50] C. Antonetti , A. M. R. Galletti , P. Accorinti , S. Alini , P. Babini , K. Raabova , E. Rozhko , A. Caldarelli , P. Righi , F. Cavani , P. Concepcion , Appl. Catal., A 2013, 466, 21–31. DOI: 10.1016/j.apcata.2013.06.023

[51] X. Y. Wu , M. V. Galkin , Z. H. Sun , K. Barta , Fully lignocellulose‐based PET analogues for the circular economy, Nat. Commun. 2022, 13, 3376. DOI: 10.1038/s41467-022-30735-4 35697677 PMC9192716

[52] Z. H. Sun , G. Bottari , A. Afanasenko , M. C. A. Stuart , P. J. Deuss , B. Fridrich , K. Barta , Nat. Catal. 2018, 1 (1), 82–92. DOI: 10.1038/s41929-017-0007-z

[53] S. Van den Bosch , W. Schutyser , R. Vanholme , T. Driessen , S. F. Koelewijn , T. Renders , B. De Meester , W. J. J. Huijgen , W. Dehaen , C. M. Courtin , B. Lagrain , W. Boerjan , B. F. Sels , Energ. Environ. Sci. 2015, 8 (6), 1748–1763. DOI: 10.1039/C5EE00204D

[54] M. V. Galkin , J. S. M. Samec , ChemSusChem 2014, 7 (8), 2154–2158. DOI: 10.1002/cssc.201402017 24910404

[55] J. Hitce , M. Crutizat , C. Bourdon , A. Vives , X. Marat , M. Dalko‐Csiba , Green Chem. 2015, 17 (7), 3756–3761. DOI: 10.1039/c5gc00759c

[56] K. H. Kim , B. A. Simmons , S. Singh , Green Chem. 2017, 19 (1), 215–224. DOI: 10.1039/c6gc02473d

[57] E. Martinez‐Hernandez , X. G. Cui , C. D. Scown , M. A. Amezcua‐Allieri , J. Aburto , B. A. Simmons , Biofuel Bioprod. Bior. 2019, 13 (4), 978–993. DOI: 10.1002/bbb.1989

[58] B. G. Harvey , C. M. Sahagun , A. J. Guenthner , T. J. Groshens , L. R. Cambrea , J. T. Reams , J. M. Mabry , ChemSusChem 2014, 7 (7), 1964–1969. DOI: 10.1002/cssc.201400019 24782220

[59] Y. H. Liao , S. F. Koelewijn , G. Van den Bossche , J. Van Aelst , S. Van den Bosch , T. Renders , K. Navare , T. Nicolai , K. Van Aelst , M. Maesen , H. Matsushima , J. M. Thevelein , K. Van Acker , B. Lagrain , D. Verboekend , B. F. Sels , Science 2020, 367 (6484), 1385–1390. DOI: 10.1126/science.aau1567 32054697

[60] D. Singh , P. T. Deota , Org. Prep. Proced. Int. 2020, 52 (4), 290–296. DOI: 10.1080/00304948.2020.1762459

[61] A. Ibrahim , Eng. Technol. Open Acc. 2018, 1 (3), 555563. DOI: 10.19080/ETOAJ.2018.01.555563

[62] M. Mascal , ACS Sustainable Chem. Eng. 2019, 7 (6), 5588–5601. DOI: 10.1021/acssuschemeng.8b06553

[63] K. Yutaka , N. Takashi , T. Takayuki , T. Kazuo , M. Yoshihisa , *Patent* , 1992. EP0267761B1

[64] P. Serna , A. Corma , ChemSusChem 2014, 7 (8), 2136–2139. DOI: 10.1002/cssc.201402061 24889545

[65] T. Maegawa , A. Akashi , K. Yaguchi , Y. Iwasaki , M. Shigetsura , Y. Monguchi , H. Sajiki , Chem. Eur. J. 2009, 15 (28), 6953–6963. DOI: 10.1002/chem.200900361 19514037

[66] https://docplayer.net/21083923‐Creating‐value‐from‐wood‐the‐borregaard‐biorefinery.html

[67] M. Fache , B. Boutevin , S. Caillol , ACS Sustainable Chem. Eng. 2016, 4 (1), 35–46. DOI: 10.1021/acssuschemeng.5b01344

[68] N. J. Gallage , B. L. Moller , Mol. Plant 2015, 8 (1), 40–57. DOI: 10.1016/j.molp.2014.11.008 25578271

[69] X. J. Shen , Q. L. Meng , Q. Q. Mei , H. Z. Liu , J. Yan , J. L. Song , D. X. Tan , B. F. Chen , Z. R. Zhang , G. Y. Yang , B. X. Han , Chem. Sci. 2020, 11 (5), 1347–1352. DOI: 10.1039/c9sc05892c PMC8148073 34123258

[70] N. J. Gallage , B. L. Moller , Mol. Plant 2015, 8 (1), 40–57. DOI: 10.1016/j.molp.2014.11.008 25578271

[71] R. J. van Putten , J. C. van der Waal , E. de Jong , C. B. Rasrendra , H. J. Heeres , J. G. de Vries , Chem. Rev. 2013, 113, 1499–1597. 10.1021/cr300182k 23394139

[72] F. J. Lai , F. Yan , P. J. Wang , S. B. Wang , S. Li , Z. T. Zhang , Chem. Eng. J. 2020, 396 (15), 125282. DOI: 10.1016/j.cej.2020.125282

[73] S. Yu , H. M. Brown , X. W. Huang , X. D. Zhou , J. E. Amonette , Z. C. Zhang , Appl. Catal., A 2009, 361 (1–2), 117–122. DOI: 10.1016/j.apcata.2009.04.002

[74] S. H. Krishna , K. F. Huang , K. J. Barnett , J. Y. He , C. T. Maravelias , J. A. Dumesic , G. W. Huber , M. De Bruyn , B. M. Weckhuysen , AIChE J. 2018, 64 (6), 1910–1922. DOI: 10.1002/aic.16172

[75] L. Hu , J. X. Xu , S. Y. Zhou , A. Y. He , X. Tang , L. Lin , J. M. Xu , Y. J. Zhao , ACS Catal. 2018, 8 (4), 2959–2980. DOI: 10.1021/acscatal.7b03530

[76] G. Li , Z. Sun , Y. Yan , Y. H. Zhang , Y. Tang , ChemSusChem 2017, 10 (3), 494–498. DOI: 10.1002/cssc.201601322 27882693

[77] L. Hu , J. X. Xu , S. Y. Zhou , A. Y. He , X. Tang , L. Lin , J. M. Xu , Y. J. Zhao , ACS Catal. 2018, 8 (4), 2959–2980. DOI: 10.1021/acscatal.7b03530

[78] J. J. Roylance , T. W. Kim , K. S. Choi , ACS Catal. 2016, 6 (3), 1840–1847. DOI: 10.1021/acscatal.5b02586

[79] C. Xu , E. Paone , D. Rodriguez‐Padron , R. Luque , F. Mauriello , Chem. Soc. Rev. 2020, 49 (13), 4273–4306. DOI: 10.1039/D0CS00041H 32453311

[80] M. Chatterjee , T. Ishizaka , H. Kawanami , Green Chem. 2014, 16 (11), 4734–4739. DOI: 10.1039/C4GC01127A

[81] G. Bottari , A. J. Kumalaputri , K. K. Krawczyk , B. L. Feringa , H. J. Heeres , K. Barta , ChemSusChem 2015, 8 (8), 1323–1327. DOI: 10.1002/cssc.201403453 25833148

[82] A. J. Kumalaputri , G. Bottari , P. M. Erne , H. J. Heeres , K. Barta , ChemSusChem 2014, 7 (8), 2266–2275. DOI: 10.1002/cssc.201402095 24924637

[83] M. Chatterjee , T. Ishizaka , H. Kawanami , Green Chem. 2014, 16 (11), 4734–4739. DOI: 10.1039/C4GC01127A

[84] K. Vikanova , E. Redina , G. Kapustin , M. Chernova , O. Tkachenko , V. Nissenbaum , L. Kustov , ACS Sustainable Chem. Eng. 2021, 9 (3), 1161–1171. DOI: 10.1021/acssuschemeng.0c06560

[85] M. Balakrishnan , E. R. Sacia , A. T. Bell , Green Chem. 2012, 14 (6), 1626–1634. DOI: 10.1039/C2GC35102A

[86] M. Tamura , K. Tokonami , Y. Nakagawa , K. Tomishige , Chem. Commun. 2013, 49 (63), 7034–7036. DOI: 10.1039/C3CC41526K 23689498

[87] A. A. Turkin , E. V. Makshina , B. F. Sels , ChemSusChem 2022, 15 (13), e202200412. DOI: 10.1002/cssc.202200412 35348300

[88] R. C. Cioc , M. Lutz , E. A. Pidko , M. Crockatt , J. C. van der Waal , P. C. A. Bruijnincx , Green Chem. 2021, 23 (1), 367–373. DOI: 10.1039/D0GC03558K PMC8327927 34381306

[89] R. C. Cioc , M. Crockatt , J. C. Waal , P. C. A. Bruijnincx , Angew. Chem., Int. Ed. 2022, 61 (17), e202114720. DOI: 10.1002/ange.202114720 PMC9304315 35014138

[90] J. J. Pacheco , M. E. Davis , PNAS 2014, 111 (23), 8363–8367. DOI: 10.1073/pnas.1408345111 24912153 PMC4060660

[91] L. Tao , T. H. Yan , W. Q. Li , Y. Zhao , Q. Zhang , Y. M. Liu , M. M. Wright , Z. H. Li , H. Y. He , Y. Cao , Chem 2018, 4 (9), 2212–2227. DOI: 10.1016/j.chempr.2018.07.007

[92] H. S. U. Chan‐Chia , S. Y. Hsueh , *Patent* , 2018. US20180057429A

[93] I. Khalil , G. Quintens , T. Junkers , M. Dusselier , Green Chem. 2020, 22 (5), 1517–1541. DOI: 10.1039/c9gc04161c

[94] A. Kayou , *Patent* , 2002. JP2002069016A

[95] Q. C. Zhu , Y. W. Song , S. Z. Guo , Z. H. Lv , *Patent* , 2008. CN101096332A

[96] R. A. F. Tomas , J. C. M. Bordado , J. F. P. Gomes , Chem. Rev. 2013, 113 (10), 7421–7469. DOI: 10.1021/cr300298j 23767849

[97] M. Sajid , X. B. Zhao , D. H. Liu , Green Chem. 2018, 20 (24), 5427–5453. DOI: 10.1039/C8GC02680G

[98] D. Y. Zhao , T. Su , Y. T. Wang , R. S. Varma , C. Len , Mol. Catal. 2020, 495, 111133. DOI: 10.1016/j.mcat.2020.111133

[99] S. Kar , Q.‐Q. Zhou , Y. Ben‐David , D. Milstein , J. Am. Chem. Soc. 2022, 144 (3), 1288–1295. DOI: 10.1021/jacs.1c10908 35007419 PMC8796234

[100] M. Mascal , ACS Sustainable Chem. Eng. 2019, 7 (6), 5588–5601. DOI: 10.1021/acssuschemeng.8b06553

[101] M. Mascal , E. B. Nikitin , Green Chem. 2010, 12 (3), 370–373. DOI: 10.1039/b918922j

[102] J. M. Carraher , P. Carter , R. G. Rao , M. J. Forrester , T. Pfennig , B. H. Shanks , E. W. Cochran , J. P. Tessonnier , Green Chem. 2020, 22 (19), 6444–6454. DOI: 10.1039/d0gc02108c

[103] I. Khalil , G. Quintens , T. Junkers , M. Dusselier , Green Chem. 2020, 22 (5), 1517–1541. DOI: 10.1039/c9gc04161c

[104] R. Lu , F. Lu , J. Z. Chen , W. Q. Yu , Q. Q. Huang , J. J. Zhang , J. Xu , Angew. Chem. Int. Ed. 2016, 55 (1), 249–253. DOI: 10.1002/anie.201509149 26592149

[105] X. G. He , Z. Y. Li , Q. D. You , Synthetic Commun. 2002, 32, 709–714. DOI: 10.1081/SCC-120002508

[106] Y. C. Hu , Z. T. Zhao , Y. T. Liu , G. Y. Li , A. Q. Wang , Y. Cong , T. Zhang , F. Wang , N. Li , Angew. Chem., Int. Ed. 2018, 57 (23), 6901–6905. DOI: 10.1002/anie.201801287 29673054

[107] S. Wang , S. Q. Ma , Q. Li , X. W. Xu , B. B. Wang , W. C. Yuan , S. H. Zhou , S. S. You , J. Zhu , Green Chem. 2019, 21, 1484–1497. DOI: 10.1039/C8GC03477J

[108] Z. J. Wei , Y. R. Cheng , K. Zhou , Y. Zeng , E. Yao , Q. Li , Y. X. Liu , Y. Sun , ChemSusChem 2021, 14 (11), 2308–2312. DOI: 10.1002/cssc.202100564 33909345

[109] Z. J. Wei , Y. R. Cheng , H. Huang , Z. H. Ma , K. Zhou , Y. X. Liu , ChemSusChem 2022, 15 (13), e202200233. DOI: 10.1002/cssc.202200233 35225422

[110] H. F. Qi , F. Liu , L. L. Zhang , L. Li , Y. Su , J. Y. Yang , R. Hao , A. Q. Wang , T. Zhang , Green Chem. 2020, 22 (20), 6897–6901. DOI: 10.1039/D0GC02280B

[111] A. Ryo , K. Tomoaki , *Patent* , 2017. US2020181105A1

[112] T. Komanoya , T. Kinemura , Y. Kita , K. Kamata , M. Hara , J. Am. Chem. Soc. 2017, 139 (33), 11493–11499. DOI: 10.1021/jacs.7b04481 28759206

[113] K. Zhou , R. H. Xie , M. T. Xiao , D. R. Guo , Z. D. Cai , S. M. Kang , Y. J. Xu , J. J. Wei , ChemCatChem 2021, 13 (8), 2074–2085. DOI: 10.1002/cctc.202001922

[114] Y. M. Xu , X. Q. Jia , J. P. Ma , J. Gao , F. Xia , X. F. Li , J. Xu , Green Chem. 2018, 20 (12), 2697–2701. DOI: 10.1039/C8GC00947C

