# Supplementary material for: A Diamine‐Oriented Biorefinery Concept Using Ammonia and Raney Ni as a Multifaceted Catalyst
Source: Chem Ing Tech. 2022 Sep 19;94(11):1808–17. doi: 10.1002/cite.202200091 (PMC9826469; doi:10.1002/cite.202200091)
Supplement: Supplementary file 1 — Supplementary Information [file CITE-94-1808-s001.pdf]

Supporting Information

A diamine-oriented biorefinery concept using ammonia and Raney Ni as a multifaceted catalyst

Xianyuan Wu[a], Mario De bruyn[b], and Katalin Barta*[a,b]

DOI: 10.1002/cite.202200091

[a]Stratingh Institute for Chemistry, University of Groningen, Groningen, The Netherlands.

[b]Department of Chemistry, Organic and Bioorganic Chemistry, University of Graz, Heinrichstrasse 28/II, 8010 Graz, Austria

*Correspondence to: katalin.barta@uni-graz.at

Table of contents

1. General information 2

2. Catalytic direct amination of biomass-derived diols to diamines 3

2.1 Reaction conditions, GC-FID traces and mass spectrographs of the crude (di)amination products 3

1. General information

Gas Chromatography (GC) was used for product identification as well as the determination of conversion and selectivity values. Product identification was performed by a GC-MS (Model: 5975C MSD) equipped with an HP-5 MS column, and helium as the carrier gas. Temperature program: 50 °C for 5 min - 10 °C·min^{-1} to 325 °C - hold for 5 min. Conversion and product selectivities were determined by GC-FID (Agilent 8890 GC) equipped with an HP-5MS column and using nitrogen as the carrier gas.

The catalytic direct amination of diols to diamines over Raney Ni catalysts with ammonia gas

The catalytic direct amination of diols into diamines was performed in 10 mL high pressure autoclaves, equipped with a magnetic stirring bar. Typically, a 4 ml glass vial was charged with 50 mg Raney Ni, 0.5 mmol diol, 2.5 mL *t*-amyl alcohol and 10 mg dodecane as an internal standard. Then the vial was sealed inside the autoclave and pressurized with 7 bar NH_3. The reactor was heated and stirred at 400 rpm for 18 h. After completion of the reaction, the reactor was cooled down to room temperature. Then, 0.1 mL solution was collected through a syringe and injected to a GC-MS or GC-FID after filtration through a PTFE filter (0.45 μm).

For catalytic amination of diols to diamines:

$$\text{Conversion (\%)} = \frac{\text{Mol of (original diols} - \text{remaining diols)}}{\text{mol of original diols}} \times 100\%$$

$$\text{Selectivity (\%)} = \frac{\text{Mol of the obtained diamine}}{\text{Mol of (original diols} - \text{remaining diols)}} \times 100\%$$

$$\text{Yield (\%)} = \text{Conversion (\%)} \times \text{Selectivity (\%)}$$

2. Catalytic direct amination of biomass-derived diols to diamines

2.1 Reaction conditions, GC-FID traces and mass spectrographs of the crude (di)amination products

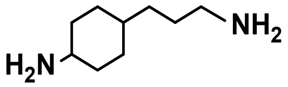

L-DA1

Reaction conditions: 0.5 mmol **L-1**, 50 mg Raney Ni, 2.5ml *t*-amyl alcohol, 7 bar NH_3, 150 °C, 18 h. After reaction, crude product was analyzed by GC-FID/MS. The yield to **L-DA1** was determined by GC-FID.

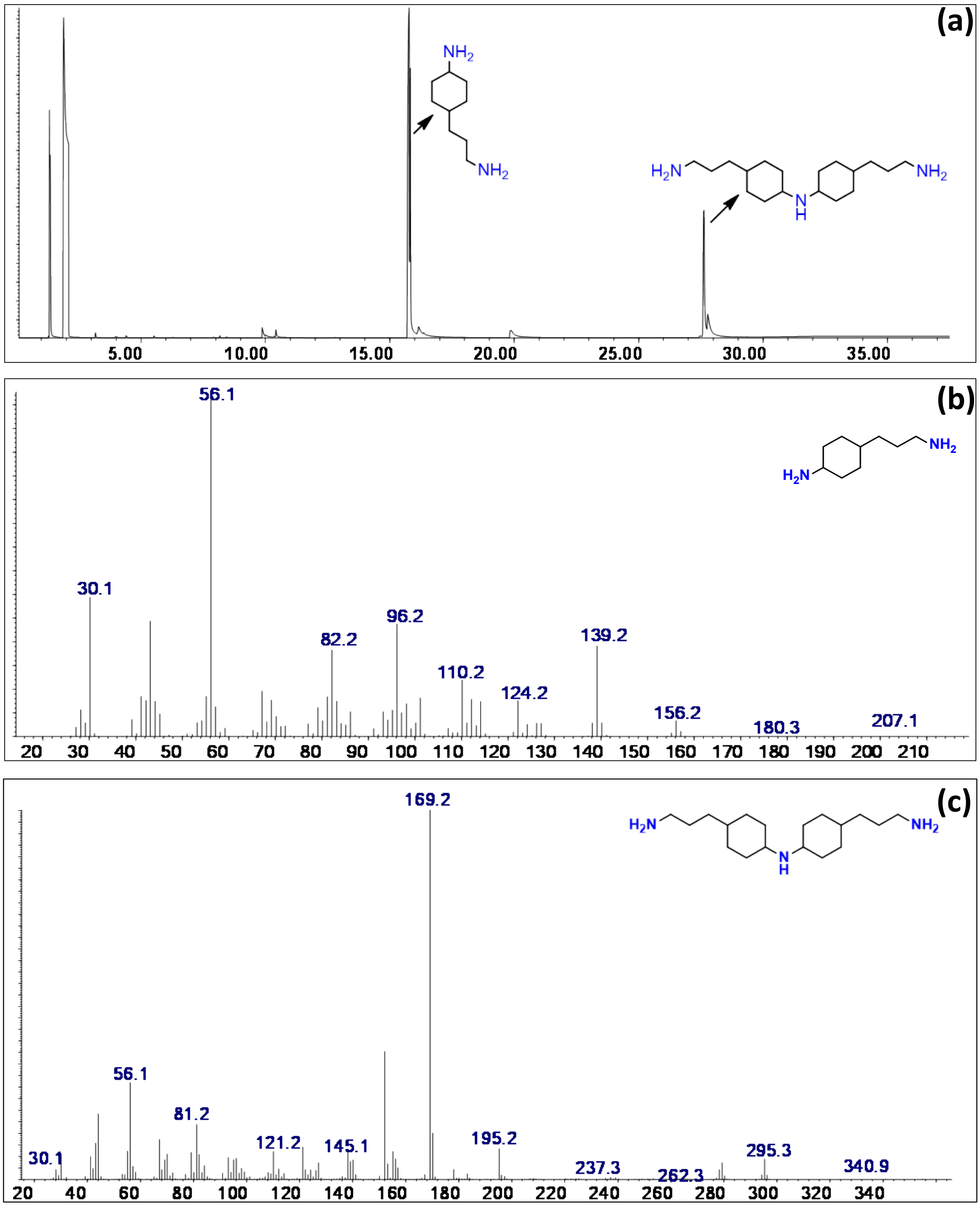

Fig. S1 GC traces of (a) crude **L-DA1** obtained by amination of **L-1**; (b) GC-MS spectrum of **L-DA1**; (c) GC-MS spectrum of **L-DA 1** dimer

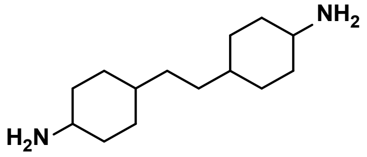

L-DA2

Reaction conditions: 0.5 mmol **L-2**, 50 mg Raney Ni, 2.5 mL *t*-amyl alcohol, 170 °C, 18 h. After reaction, catalyst was separated by filtration and *t*-amyl alcohol solvent was removed under reduced pressure. Finally, crude product (107.4 mg) was obtained in an isolated yield of 95.7 %. The crude product (**L-DA2**) was characterized by GC-FID/MS.

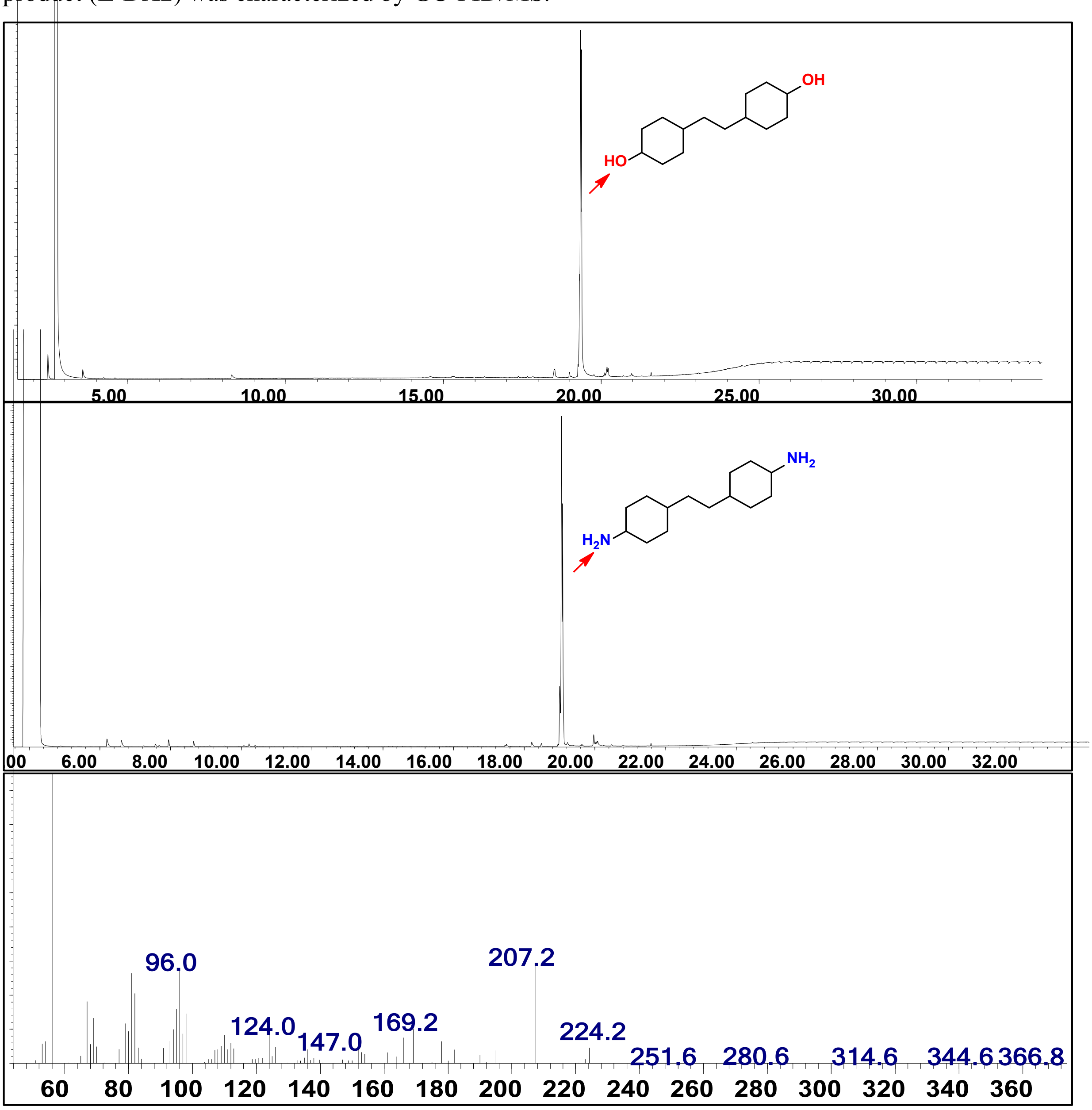

Fig. S2. GC traces of (a) pure **L-2**; (b) crude **L-DA2** obtained by amination of **L-2**; (c) GC-MS spectrum of **L-DA2**

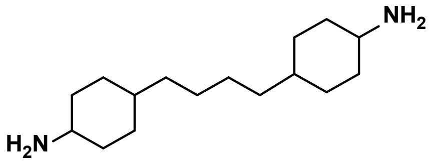

L-DA3

Reaction conditions: 0.5 mmol **L-3**, 50 mg Raney Ni, 2.5 mL *t*-amyl alcohol, 170 °C, 18 h. After reaction, catalyst was separated by filtration and *t*-amyl alcohol solvent was removed under reduced pressure. Finally, crude product (123.7 mg) was obtained in an isolated yield of 98 %. The crude product (**L-DA3**) was characterized by GC-FID/MS.

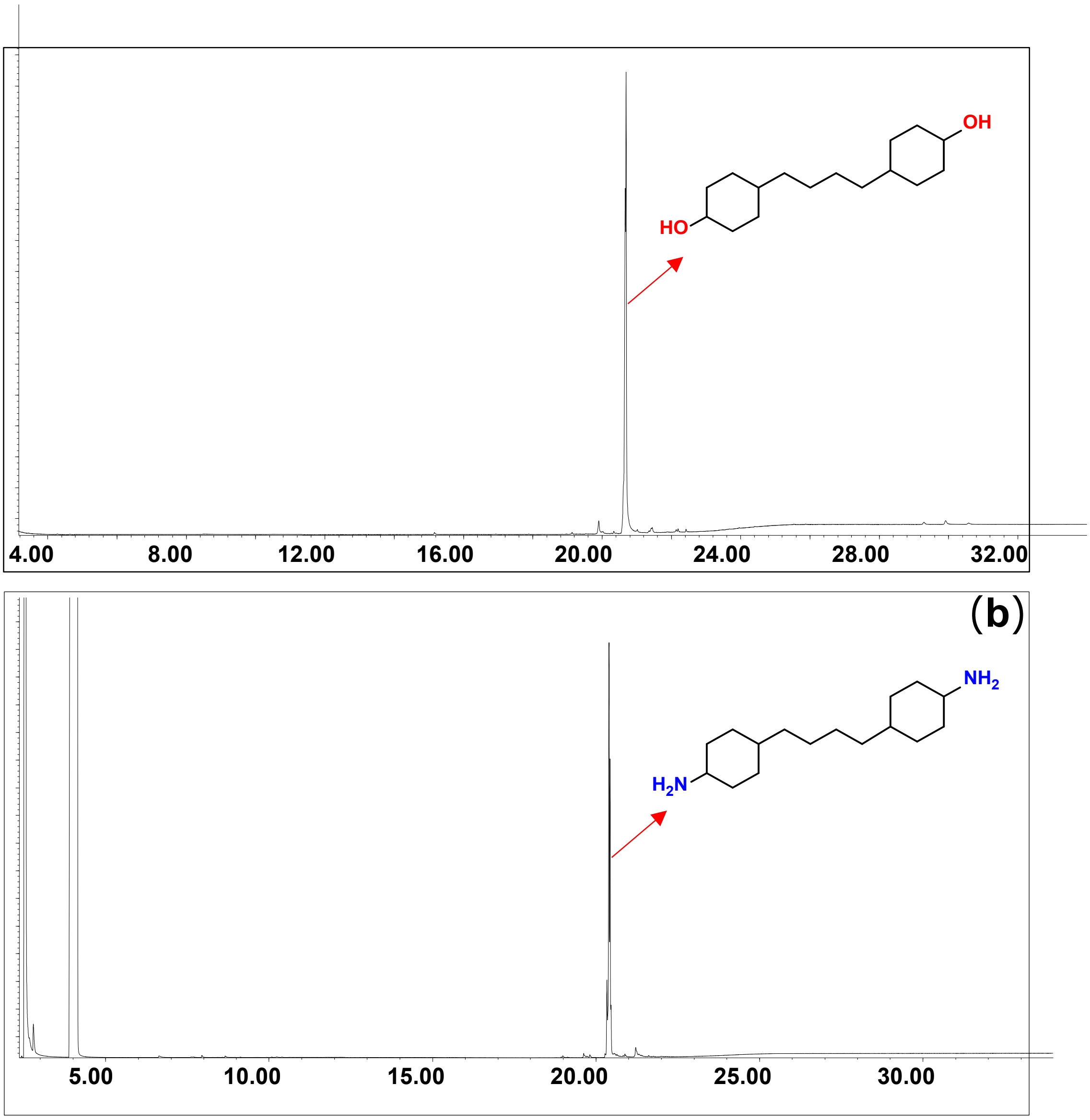

Fig. S3. GC traces of (a) pure **L-3**; (b) crude **L-DA3** obtained by amination of **L-3**

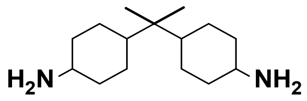

L-DA4

Reaction conditions: 0.5 mmol **L-4** diol, 50 mg Raney Ni, 2.5 mL *t*-amyl alcohol, 170 °C, 18 h. After reaction, catalyst was separated by filtration and *t*-amyl alcohol solvent was removed under reduced pressure. Finally, crude product (115.6 mg) was obtained in an isolated yield of 97.0 %. The crude product (**L-DA4**) was characterized by GC-FID/MS.

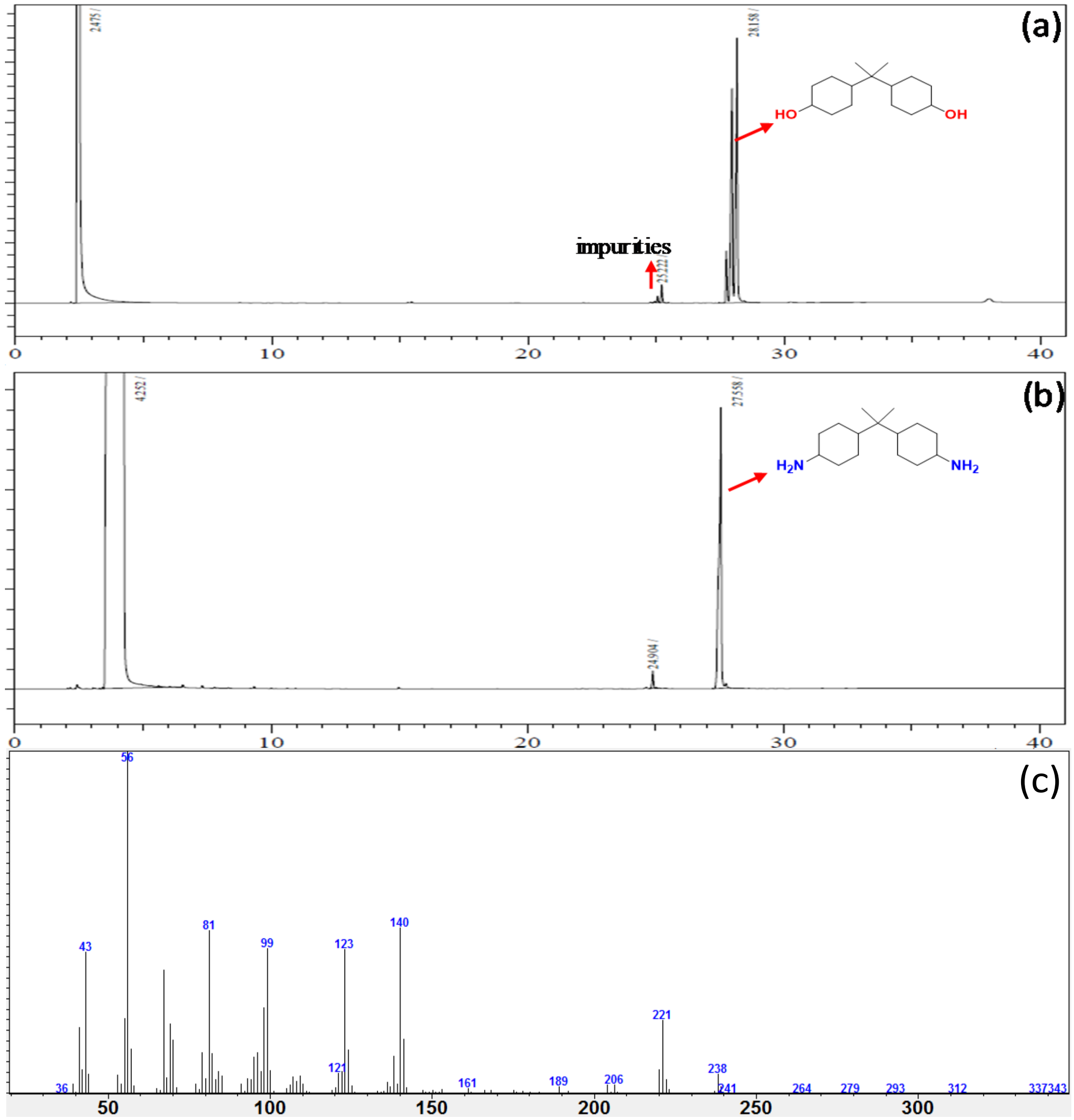

Fig. S4. GC traces of (a) pure **L-4**; (b) crude **L-DA4** obtained by amination of **L-4**; (c) GC-MS spectrum of **L-DA4**

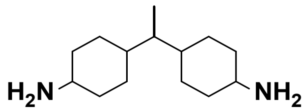

L-DA5

Reaction conditions: 0.5 mmol **L-5**, 50 mg Raney Ni, 2.5 mL *t*-amyl alcohol, 170 °C, 18 h. After reaction, catalyst was separated by filtration and *t*-amyl alcohol solvent was removed under reduced pressure. Finally, crude product (108.6 mg) was obtained in an isolated yield of 96.8 %. The crude product (**L-DA5**) was characterized by GC-FID/MS.

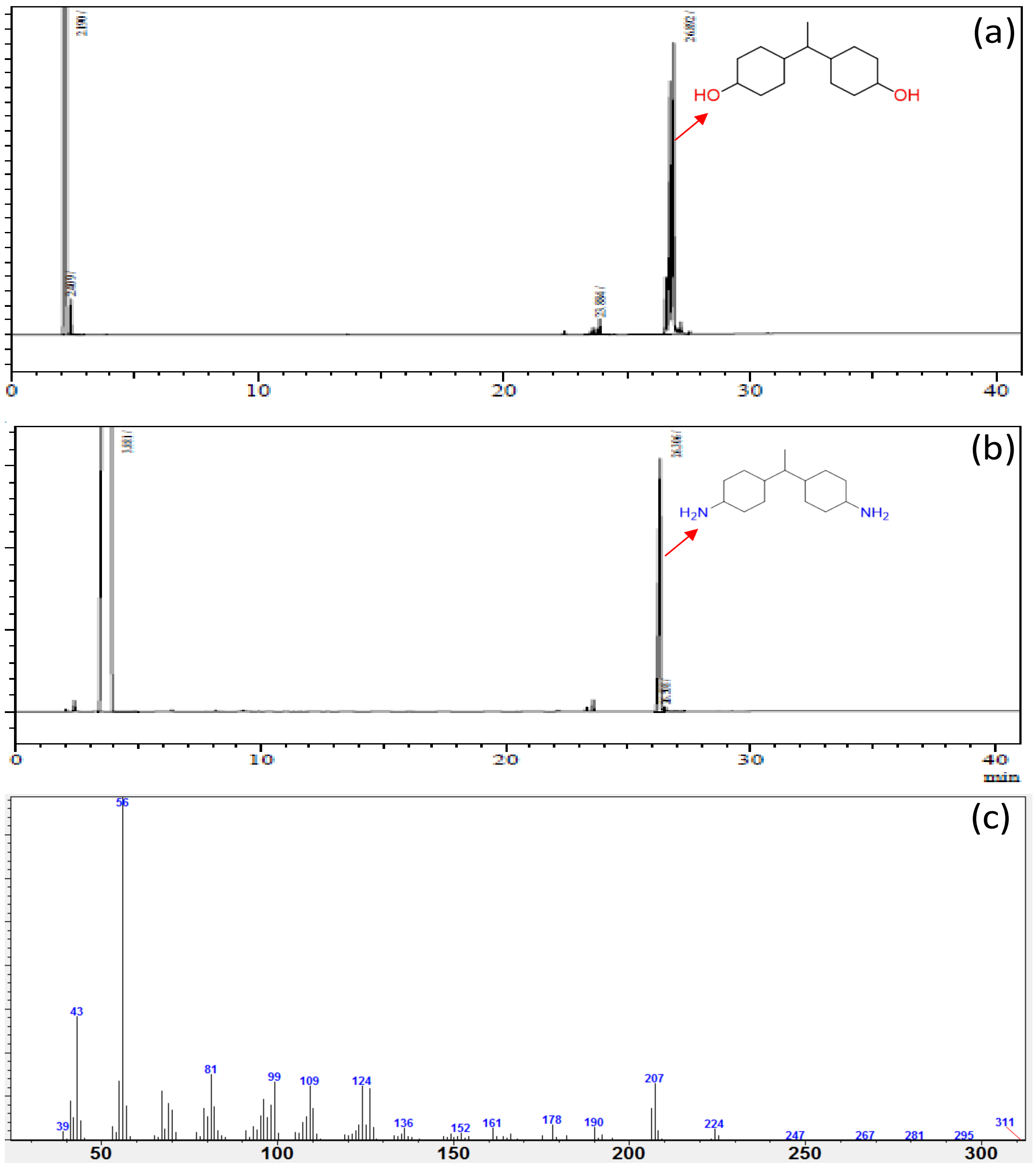

Fig. S5. GC traces of (a) pure **L-5**; (b) crude **L-DA5** obtained by amination of **L-5**; (c) GC-MS spectrum of **L-DA5**

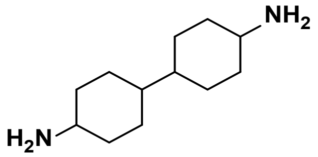

L-DA6

Reaction conditions: 0.5 mmol **L-6**, 50 mg Raney Ni, 2.5 mL *t*-amyl alcohol, 170 °C, 18 h. After reaction, catalyst was separated by filtration and *t*-amyl alcohol solvent was removed under reduced pressure. Finally, crude product (94.7 mg) was obtained in an isolated yield of 96.5 %. The crude product (**L-DA6**) was characterized by GC-FID/MS.

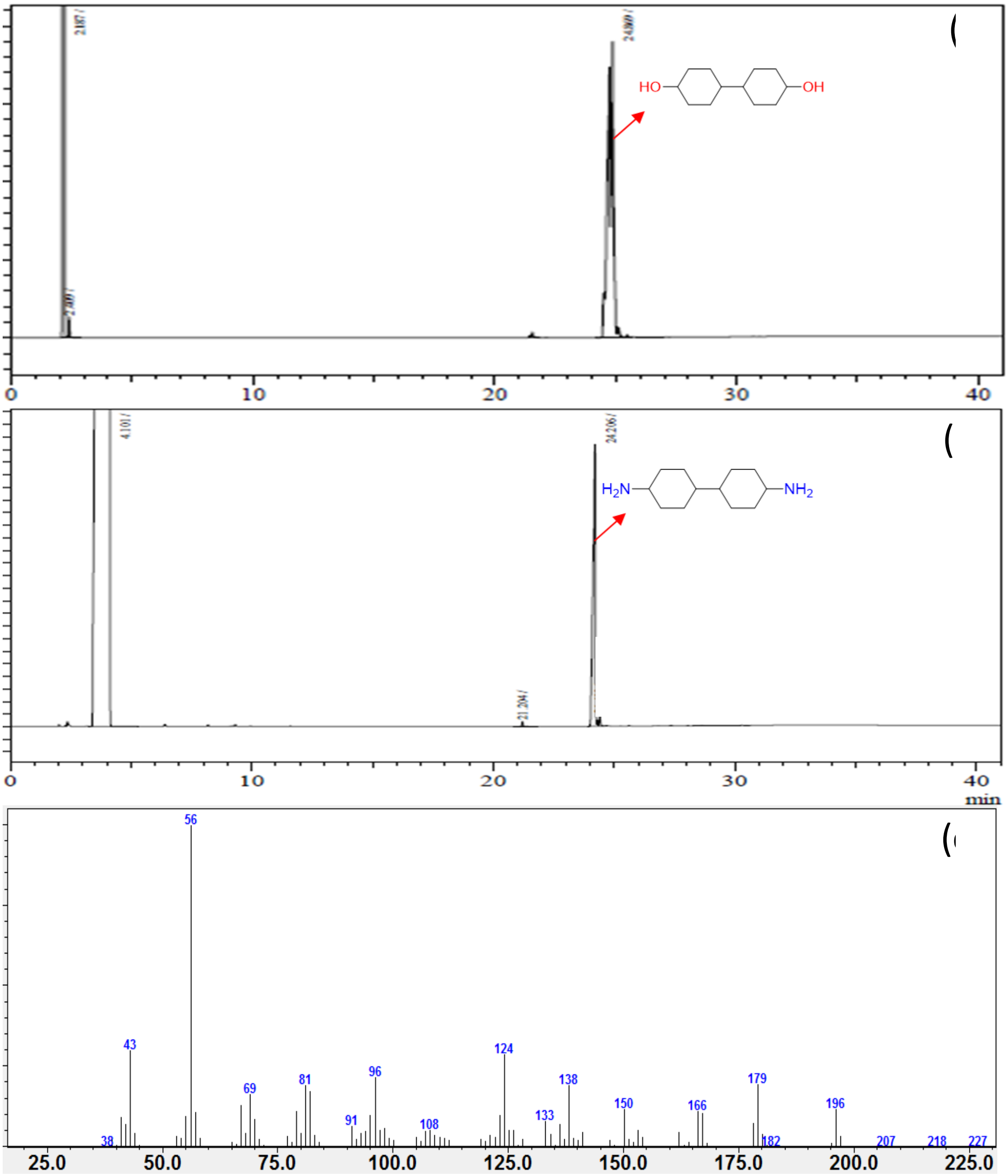

Fig. S6. GC traces of (a) pure **L-6**; (b) crude **L-DA6** obtained by amination of **L-6**; (c) MS spectrum of **L-DA6**

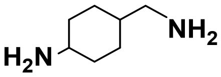

L-DA7

Reaction conditions: 0.5 mmol **L-7**, 50 mg Raney Ni, 2.5ml *t*-amyl alcohol, 7 bar NH_3, 140 °C, 18 h. After reaction, crude product was analyzed by GC-FID/MS and the yield to **L-DA7** was determined by GC-FID.

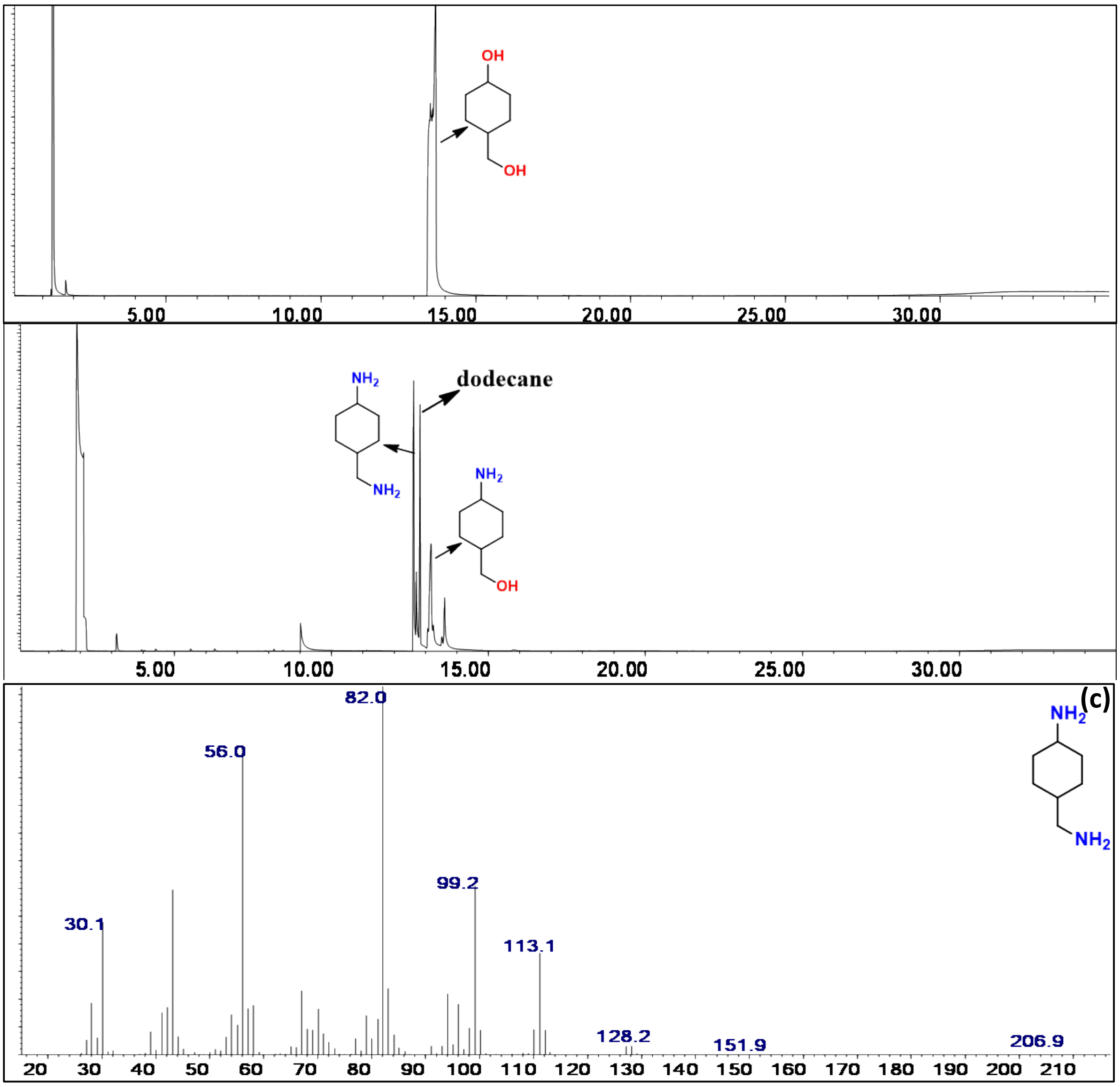

Fig. S7. GC traces of (a) pure **L-7**; (b) crude **L-DA7** obtained by amination of **L-7**; (c) MS spectrum of **L-DA7**

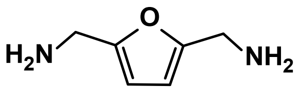

C-DA1

Reaction conditions: 0.5 mmol **C-1**, 50 mg Raney Ni, 2.5 mL *t*-amyl alcohol, 160-180 °C, 18 h. After reaction, crude product was analyzed GC-FID/MS and the yield to **C-DA1** was determined by GC-FID.

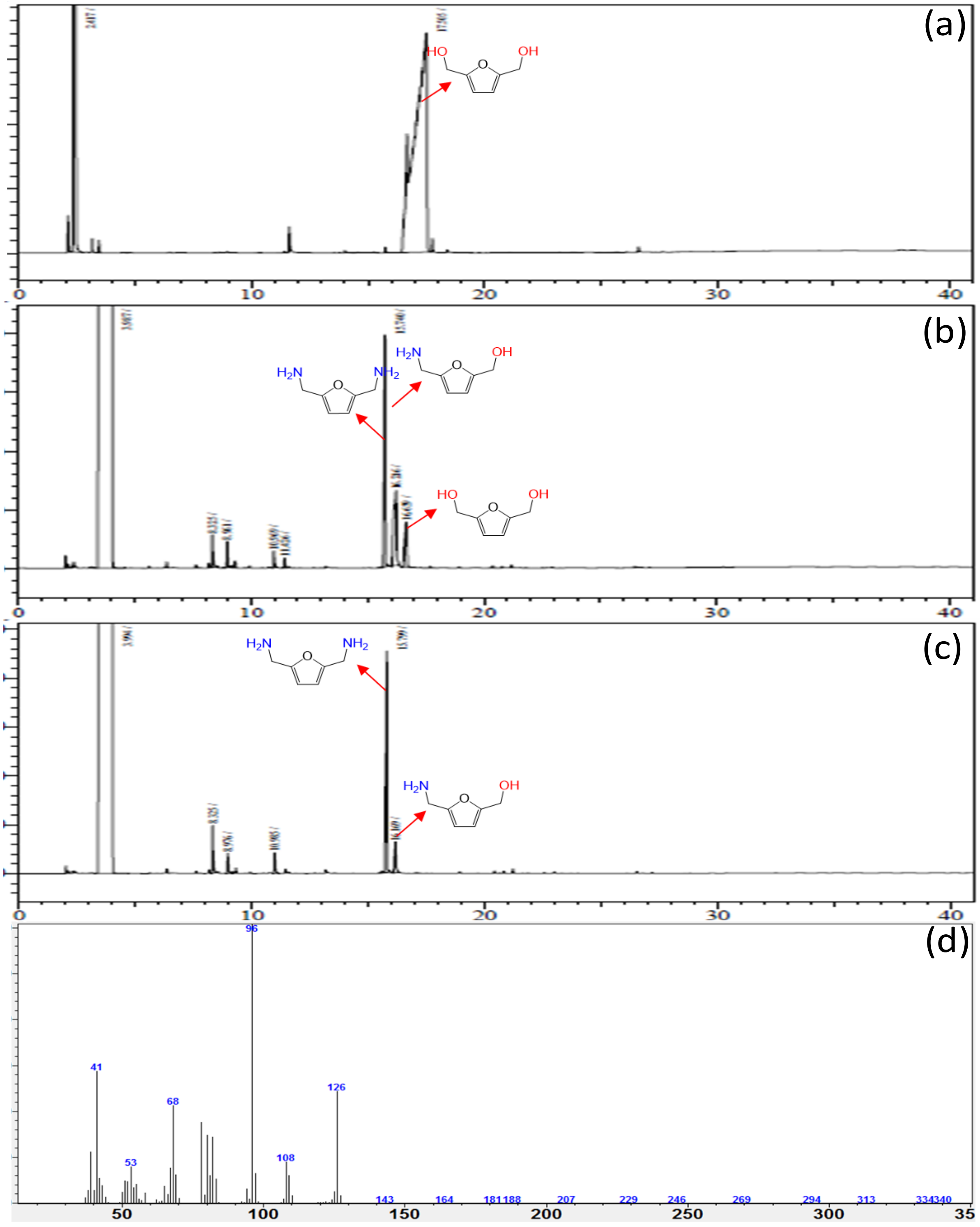

Fig. S8. GC traces of pure **C-1**; crude **C-DA1** obtained by amination of **C-1** at a) 160 °C, b) 170 °C, c) 180 °C; (d) MS spectrum of **C-DA1**

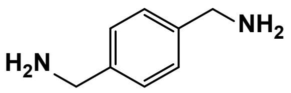

C-DA 2

Reaction conditions: 0.5 mmol **C-2**, 50 mg Raney Ni catalyst, 2.5 mL *t*-amyl alcohol, 10 mg dodecane, 7 bar NH_3, 160 °C, 18 h. After reaction, crude product was analyzed by GC-FID/MS and the yield to **C-DA2** was determined based the calibration curve using dodecane as internal standard.

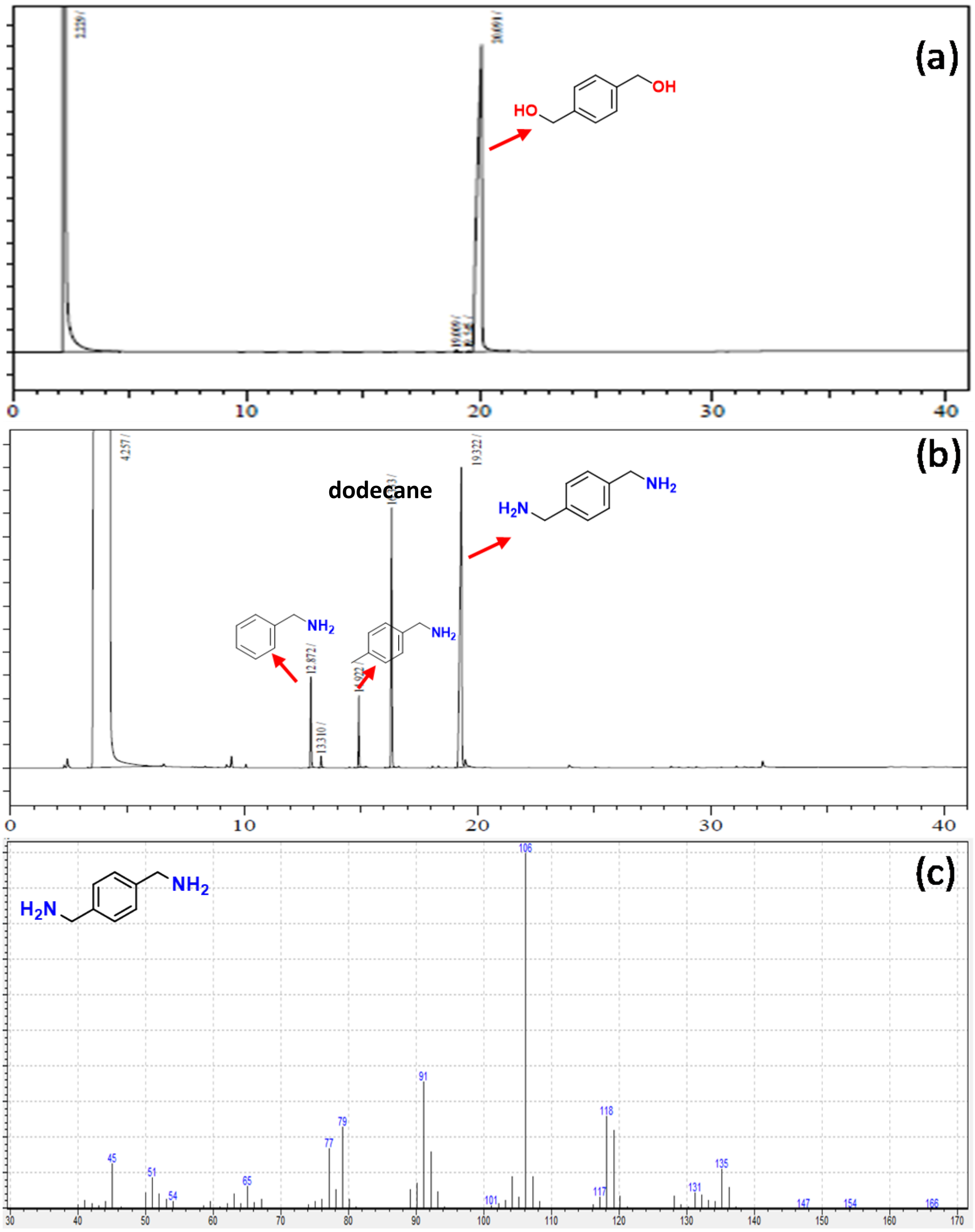

Fig. S9 GC traces of (a) pure **C-2**; (b) crude **C-DA2** obtained by amination of **C-2**; (c) MS spectrum of **C-DA2**

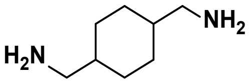

C-DA 3

Reaction conditions: 0.5 mmol **C-3**, 50 mg Raney Ni, 2.5 mL *t*-amyl alcohol, 7 bar NH_3, 160 °C, 18 h, 10 mg dodecane. After reaction, crude product was analyzed by GC-FID and the yield to **C-DA3** was determined based the calibration curve using dodecane an internal standard.

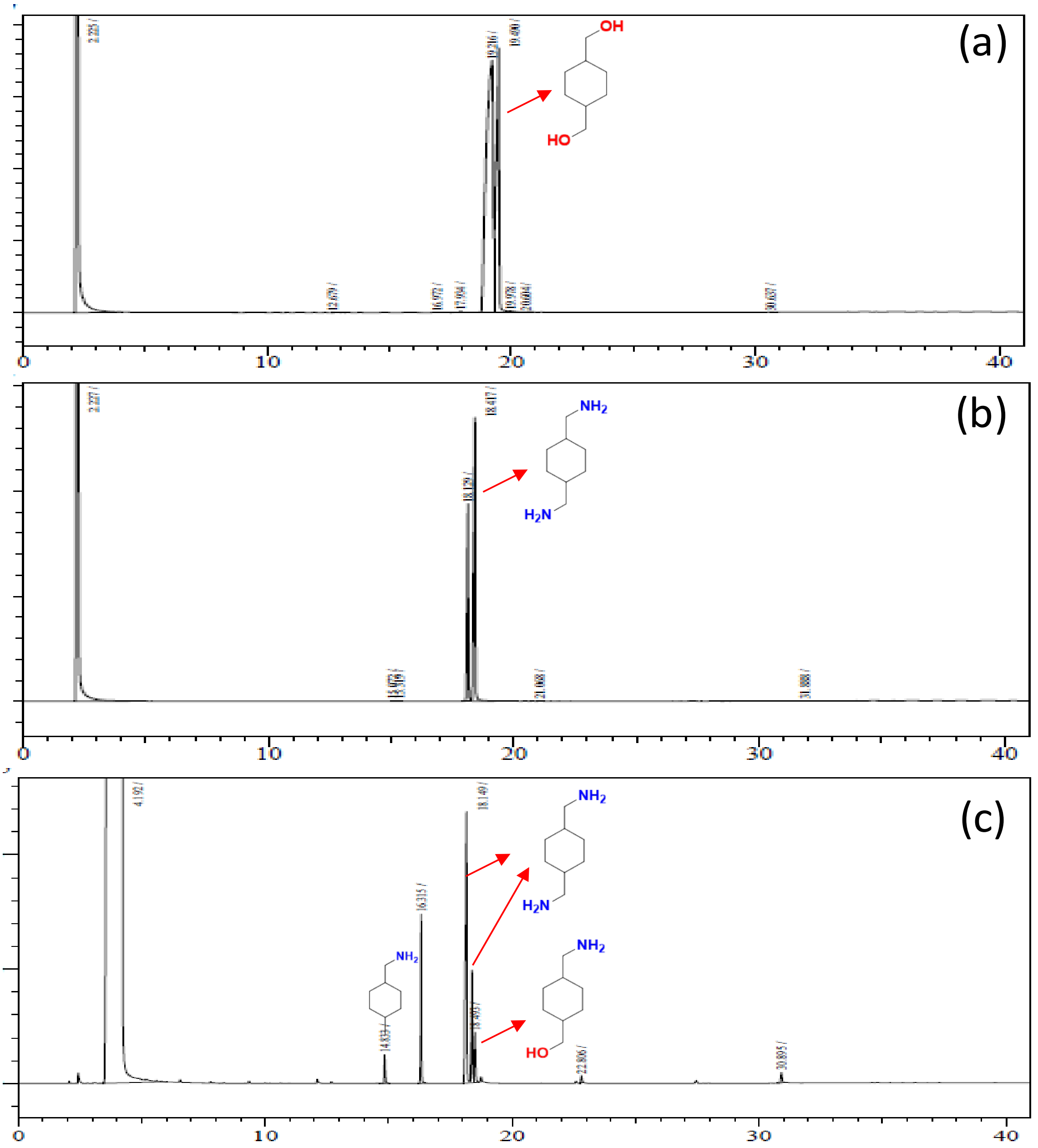

Fig. S10 GC traces of (a) pure **C-3**; (b) crude **C-DA3** obtained by amination of **C-3**; (c) MS spectrum of **C-DA3**
